# Minimum conditions for accurate modeling of urea production via co-electrolysis

**DOI:** 10.1038/s42004-023-00990-7

**Published:** 2023-09-13

**Authors:** Ricardo Urrego-Ortiz, Santiago Builes, Francesc Illas, Stefan T. Bromley, Marta Costa Figueiredo, Federico Calle-Vallejo

**Affiliations:** 1 Departament de Ciència de Materials i Química Física & Institut de Química Teòrica i Computacional (IQTCUB), Universitat de Barcelona, C/ Martí i Franquès 1, 08028 Barcelona, Spain; 2 Nano-Bio Spectroscopy Group and European Theoretical Spectroscopy Facility (ETSF), Department of Polymers and Advanced Materials: Physics, Chemistry and Technology, University of the Basque Country UPV/EHU, Av. Tolosa 72, 20018 San Sebastián, Spain; 3 Escuela de Ciencias Aplicadas e Ingeniería, Universidad EAFIT, Carrera 49 # 7 sur 50, 050022 Medellín, Colombia; 4 Institució Catalana de Recerca i Estudis Avançats (ICREA), Passeig Lluís Companys 23, 08010 Barcelona, Spain; 5 Eindhoven Institute of Renewable Energy Systems (EIRES), Eindhoven University of Technology, PO Box 513, Eindhoven, 5600 MB The Netherlands; 6 IKERBASQUE, Basque Foundation for Science, Plaza de Euskadi 5, 48009 Bilbao, Spain

**Keywords:** Electrocatalysis, Density functional theory, Computational chemistry

## Abstract

Co-electrolysis of carbon oxides and nitrogen oxides promise to simultaneously help restore the balance of the C and N cycles while producing valuable chemicals such as urea. However, co-electrolysis processes are still largely inefficient and numerous knowledge voids persist. Here, we provide a solid thermodynamic basis for modelling urea production via co-electrolysis. First, we determine the energetics of aqueous urea produced under electrochemical conditions based on experimental data, which enables an accurate assessment of equilibrium potentials and overpotentials. Next, we use density functional theory (DFT) calculations to model various co-electrolysis reactions producing urea. The calculated reaction free energies deviate significantly from experimental values for well-known GGA, meta-GGA and hybrid functionals. These deviations stem from errors in the DFT-calculated energies of molecular reactants and products. In particular, the error for urea is approximately -0.25 ± 0.10 eV. Finally, we show that all these errors introduce large inconsistencies in the calculated free-energy diagrams of urea production via co-electrolysis, such that gas-phase corrections are strongly advised.

## Abstract (web summary)

Co-electrolysis of nitrogen oxides and carbon oxides has been studied for over two decades but remains largely inefficient with numerous persisting knowledge voids. Here, the authors report a thermodynamic basis for modelling urea production *via* co-electrolysis using several exchange-correlation functionals, highlighting the importance of gas-phase error assessment in computational electrocatalysis.

## Introduction

The electrochemical co-reduction of species containing nitrogen and carbon to produce chemical commodities can be carried out using renewable electricity^1–4^, simultaneously aiding to restore the severely imbalanced cycles of nitrogen and carbon^5–7^. Among the potential products, urea (CO(NH_2_)_2_) is an appealing C-N compound given its enormous relevance in modern agriculture^8–10^, and the large energy demands for its industrial production^2,9,11,12^. Although electrocatalytic urea production from N- and C-oxides has been studied at the laboratory scale for more than two decades^13–18^, critical challenges regarding the electrochemistry of the C-N coupling are yet to be solved before industrial applications are at hand^19,20^. Some of these challenges are the large associated overpotentials, elusive reaction mechanisms, and low selectivity caused by the concurrent formation of H_2_, CO, formic acid (HCOOH), ammonia (NH_3_), and other single-carbon and/or single-nitrogen species^16,21–23^.

Either in tandem with experiments or in standalone computational studies, density functional theory (DFT) methods have been extensively used to investigate the electrosynthesis of urea from N- and C-containing feedstocks and design enhanced catalysts^2,24–28^. These studies frequently use exchange-correlation (xc) functionals following the generalized gradient approximation (GGA), as they provide a reasonable tradeoff between computational cost and accuracy for the properties of molecules and surfaces^29–32^. A recent example is the work of Wan et al., in which DFT-based thermodynamic and kinetic models were proposed to explain the selective C-N bond formation on Cu electrodes and rationalize the experimental observations of Shibata et al.^14,16,33^ using the BEEF-vdW functional^34^. Notwithstanding, the limitations of GGA functionals are well known for describing gaseous molecules containing multiple bonds, such as O_2_^30,35,36^, N_2_ and NO_x_^37–39^, and carbon-containing species^40–43^. These limitations can cause large discrepancies between calculated and experimental equilibrium potentials and impair the predictive capabilities of GGA-based heterogenous (electro)catalytic models, where molecules and surfaces are simultaneously involved^39,44,45^.

Approaches to overcome some of the shortcomings of GGA functionals, such as meta-GGA^46^ and hybrid functionals^47^, tend to perform better for the prediction of gas-phase thermochemistry. Unlike GGA functionals, meta-GGA functionals include an approximate dependence on the kinetic energy density^48^, while hybrid functionals incorporate a proportion of exact nonlocal Fock exchange^49–52^. Interestingly, previous works have shown that when GGA, meta-GGA, and/or hybrid functionals are used to model various families of C- ^40,43^ and N-containing compounds^38,39,53^, H_2_O_2(g)_ and O_2(g)_^36,44,45^, sizable gas-phase errors are still found. Such errors are systematic and can be mitigated by means of inexpensive semiempirical corrections^38,40,41,43,53^. This strongly suggests that a cautious and early assessment of gas-phase errors is needed to guarantee the accuracy of (electro)catalytic models based upon DFT calculations.

Herein, we study the co-electrolysis of different nitrogen (N_2(g)_, NO_(g)_, ${{NO}}_{3\left({aq}\right)}^{-}$) and carbon oxides (CO_(g)_, CO_2(g)_) as feedstocks to produce aqueous urea (CO(NH_2_)_2(aq)_) using several exchange-correlation functionals: four GGA functionals, two meta-GGA functionals, and two hybrid functionals. For most gas-phase compounds under study at these three levels of DFT, we pinpoint and correct large gas-phase errors in the calculated energies. Our results stress the importance of gas-phase error assessment in computational electrocatalysis and provide an accurate starting point for modeling urea production on real catalysts by co-electrolysis of CO_x_ and NO_x_ feedstocks.

## Methodology

### Computational methods

The Vienna ab initio simulation package (VASP)^54^ was used to perform the DFT calculations of H_2(g)_, N_2(g)_, O_2(g)_, H_2_O_(g)_, NH_3(g)_, CO(NH_2_)_2(g)_, CO_(g)_, CO_2(g)_, NO_(g)_, HNO_3(g)_, and C_(s)_. All compounds were modeled in their gas-phase in boxes of 15 × 15 × 15 Å^3^ (in some cases, we changed the size of the vectors by ±0.1 Å to see if more negative energies were found, which was the case only for NO). C_(s)_ was represented here by graphene as a reasonable DFT model of graphite. The latter approximation is enabled by the fact that the interlayer cohesive energy of graphite is small (0.031–0.064 eV)^55–59^. The calculations were carried out for a range of DFT functionals ascending the so-called “Jacob’s ladder”^60^: namely GGAs (PBE^61^, PW91^62^, RPBE^63^, BEEF-vdW^64^), meta-GGAs (TPSS^48^, SCAN^65^), and hybrids (PBE0^66^, B3LYP^67^). The C-C distances for graphene obtained in all cases were close to the experimental value of 1.42 Å (PBE: 1.43 Å, PW91: 1.43 Å, RPBE: 1.43 Å, BEEF-vdW: 1.43 Å, TPSS: 1.42 Å, SCAN: 1.42 Å, B3LYP: 1.42 Å, PBE0: 1.42 Å)^68^. The projector augmented-wave (PAW) method was used to represent the interactions between core electron density and valence electrons^69^. A plane-wave cutoff of 450 eV was used in all calculations, assuring converged Δ${ZPE}$ and reaction energies for the gaseous urea production from N_2(g)_ and CO_2(g)_ using PBE (${N}_{2\left(g\right)}+{{CO}}_{2\left(g\right)}+{3H}_{2\left(g\right)}\to {{{CO}\left(N{H}_{2}\right)}_{2}}_{\left(g\right)}+{H}_{2}{O}_{\left(g\right)}$). In fact, the difference between the reaction energy and Δ${ZPE}$ obtained with this cutoff differed only by ~0.01 eV from those obtained with a tighter cutoff of 1000 eV (see Supplementary Fig. 1 and Supplementary Table 1 in Supplementary Note 1). The geometry of each molecule was relaxed using the conjugate gradient algorithm until the final forces between the atoms were lower than 0.01 eV Å^−1^. Gaussian smearing with an electronic temperature of 10^−3^ eV was used to ease the convergence of the self-consistent field procedure and, upon convergence, all energies were extrapolated to 0 K. Since the code used is intrinsically periodic, the calculations for molecules were carried out at the Γ-point. Conversely, for graphene a Monkhorst-Pack grid^70^ of 8 × 8 × 1 special k-points was used. Spin-unrestricted calculations were performed for O_2(g)_ (triplet) and NO_(g)_ (doublet). Further details of the input files used to perform the calculations are provided in Supplementary Note 7 and the coordinates of the converged geometries are given in Supplementary Note 8.

The thermodynamic analyses in this study are based upon free energies. The free energy of compound *i* (${G}_{i}^{{DFT}}$) is approximated by means of its DFT energy (${E}_{i}^{{DFT}}$), the calculated zero-point energy (${{ZPE}}_{i}$) using harmonic frequencies, the difference between the formation enthalpies at 298.15 and 0 K (${\Delta }_{f}{H}_{{i\; @}298.15{K}}-{\Delta }_{f}{H}_{i@0{K}}$), and the entropic contributions ($T{S}_{i}$) taken from thermodynamic tables at *T* = 298.15 K, as shown in Eq. 1^71–75^. Supplementary Table 2 compiles the DFT-calculated energies, ZPEs, experimental TS, and the differences between the experimental formation enthalpies between 0 and 298.15 K for the compounds under study (see also Supplementary Table 5). We provide more details of the thermal contributions in Supplementary Note 4.

1 $${G}_{i}^{{DFT}}\approx {E}_{i}^{{DFT}}+{{ZPE}}_{i}+({\Delta }_{f}{H}_{i{{{{{\rm{@}}}}}}298.15K}-{\Delta }_{f}{H}_{i{{{{{\rm{@}}}}}}0K})-T{S}_{i}$$

### Co-electrolysis modeling

We consider the six reactions shown below in which urea is produced by the simultaneous reduction of different C- and N-containing species.

2 $${N}_{2\left(g\right)}+{{CO}}_{\left(g\right)}+{4H}^{+}+{4e}^{-}\to {{{CO}\left(N{H}_{2}\right)}_{2}}_{\left({aq}\right)}$$

3 $${N}_{2\left(g\right)}+{{CO}}_{2\left(g\right)}+{6H}^{+}+{6e}^{-}\to {{{CO}\left(N{H}_{2}\right)}_{2}}_{\left({aq}\right)}+{H}_{2}{O}_{\left(l\right)}$$

4 $${2{NO}}_{\left(g\right)}+{{CO}}_{\left(g\right)}+{8H}^{+}+{8e}^{-}\to {{{CO}\left(N{H}_{2}\right)}_{2}}_{\left({aq}\right)}+2{H}_{2}{O}_{\left(l\right)}$$

5 $${2{NO}}_{\left(g\right)}+{{CO}}_{2\left(g\right)}+{10H}^{+}+{10e}^{-}\to {{{CO}\left(N{H}_{2}\right)}_{2}}_{\left({aq}\right)}+3{H}_{2}{O}_{\left(l\right)}$$

6 $${2{NO}}_{3\left({aq}\right)}^{-}+{{CO}}_{\left(g\right)}+{16H}^{+}+{14e}^{-}\to {{{CO}\left(N{H}_{2}\right)}_{2}}_{\left({aq}\right)}+6{H}_{2}{O}_{\left(l\right)}$$

7 $${2{NO}}_{3\left({aq}\right)}^{-}+{{CO}}_{2\left(g\right)}+{18H}^{+}+{16e}^{-}\to {{{CO}\left(N{H}_{2}\right)}_{2}}_{\left({aq}\right)}+7{H}_{2}{O}_{\left(l\right)}$$

**Fig. 1:**
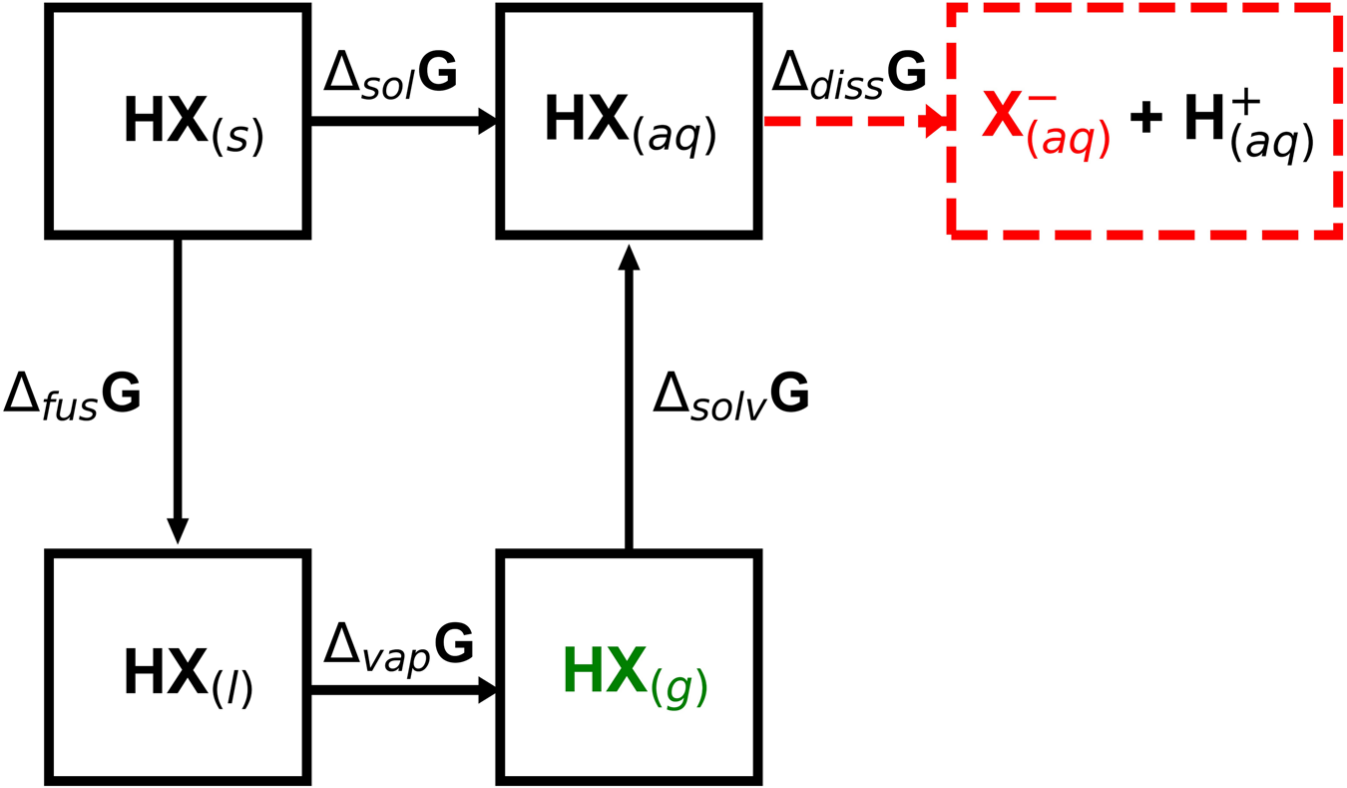
Thermodynamic framework. Scheme relating the energy differences between the states of a generic compound HX. The subindices s, l, and g represent HX in the solid, liquid, and gas phases, respectively. The total energy of HX in the gas phase (in green) can be estimated from DFT, using Eq. 1. The subindex aq refers to hydrated HX, i.e, HX_(s)_ surrounded by water. In red, an anion is produced from the dissociation of HX_(aq)_. ${\Delta }_{{sol}}G$ is the free energy of solution (the energy associated to the dissolution of one mole of HX_(s)_ in an infinite amount of water); ${\Delta }_{{fus}}G$ is the fusion free energy; ${\Delta }_{{vap}}G$ is the vaporization energy; ${\Delta }_{{solv}}G$ is the solvation free energy, defined as the energy required to bring a mole of HX_(g)_ from vacuum to a water reservoir; ${\Delta }_{{diss}}G$ is the dissociation free energy in solution. The dashed red lines indicate that dissociation occurs only for HNO_3_ in this study.

These reactions involve reactants and products in different physical states, such that, under the appropriate external potential, gaseous and aqueous compounds react to produce hydrated urea and liquid water. As DFT simulations of liquids and aqueous systems are possible but challenging and time-consuming and additional statistical analyses are necessary, the DFT values of the corresponding gas-phase references, which are rapidly calculated, serve as the basis to estimate their energetics via semiempirical considerations, as depicted in Fig. 1. Moreover, as computational solvation methods have discrepancies with respect to experiments^76^ that are larger than the accuracies of the experimental measurements, we do not expect that calculating the solvation energies of the species using DFT would yield lower errors.

The scheme in Fig. 1 shows the energy differences between the states of a generic compound HX. Figure 1 along with the additional considerations detailed below were used to calculate the free energies of all the compounds in the co-electrolysis reactions. We note that thermodynamic cycles based on experimental equilibrium potentials have also been used to semiempirically obtain the free energies of ionic species from the DFT-calculated energies of neutral solids and gaseous compounds^37,77,78^. In the Supplementary Note 6, we show in a stepwise fashion how the free energy of nitrate can be estimated using this approach.

(i) The energetics of proton-electron pairs was calculated by means of the computational hydrogen electrode, which is based on the following equilibrium in solution: ${H}^{+}+{e}^{-}\leftrightarrow \frac{1}{2}{H}_{2\left(g\right)}$, such that $\frac{1}{2}{\mu }_{{H}_{2\left(g\right)}}^{0}={\mu }_{\left({H}^{+}+{e}^{-}\right)}^{0}$^79^.
(ii) Based on Fig. 1, the free energy of formation of aqueous urea $({{\Delta }_{f}G}_{{{CO}\left(N{H}_{2}\right)}_{2\left({aq}\right)}}^{0})$ was estimated by adding the experimental solvation energy $({\Delta }_{{solv}}{G}_{{{CO}\left(N{H}_{2}\right)}_{2\left({aq}\right)}}^{\exp })$ to the DFT-calculated formation energy of gaseous urea $({{\Delta }_{f}G}_{{{CO}\left(N{H}_{2}\right)}_{2\left(g\right)}}^{{DFT}})$, i.e, ${{\Delta }_{f}G}_{{{CO}\left(N{H}_{2}\right)}_{2\left({aq}\right)}}^{0}={{\Delta }_{f}G}_{{{CO}\left(N{H}_{2}\right)}_{2\left(g\right)}}^{{DFT}}+{\Delta }_{{solv}}{G}_{{{CO}\left(N{H}_{2}\right)}_{2\left({aq}\right)}}^{\exp }$. The experimental solvation energy can be obtained as the difference between the experimental formation energy of aqueous urea $({\Delta }_{f}{G}_{{{CO}\left(N{H}_{2}\right)}_{2\left({aq}\right)}}^{\exp })$ and the experimental formation energy of gaseous urea $({\Delta }_{f}{G}_{{{CO}\left(N{H}_{2}\right)}_{2\left(g\right)}}^{\exp }=-1.57{eV})$^71,73^. ${\Delta }_{f}{G}_{{{CO}\left(N{H}_{2}\right)}_{2\left({aq}\right)}}^{\exp }$ is calculated, in accordance with Fig. 1, by combining the experimental free energy of solution $({\Delta }_{{sol}}{G}_{{{CO}\left(N{H}_{2}\right)}_{2}}^{\exp }=-0.07{eV})$^73^ and the experimental formation energy of solid urea $({\Delta }_{f}{G}_{{{CO}\left(N{H}_{2}\right)}_{2\left(s\right)}}^{\exp }=-2.04\,{eV})$^71^, thus ${\Delta }_{f}{G}_{{{CO}\left(N{H}_{2}\right)}_{2\left({aq}\right)}}^{\exp }=-2.11{eV}$. Finally, ${\Delta }_{{solv}}{G}_{{{CO}\left(N{H}_{2}\right)}_{2\left({aq}\right)}}^{\exp }= -0.54{eV}$ and ${{\Delta }_{f}G}_{{{CO}\left(N{H}_{2}\right)}_{2\left({aq}\right)}}^{0}={{\Delta }_{f}}G_{{{CO}\left(N{H}_{2}\right)}_{2\left(g\right)}}^{{DFT}}-0.54{eV}$.
(iii) Following Fig. 1, the free energy of formation of liquid water (${{\Delta }_{f}G}_{{{H}_{2}O}_{(l)}}^{0}$) was obtained by subtracting the experimental water vaporization energy $({\Delta }_{{vap}}{G}_{{H}_{2}O}^{\exp }=0.09{eV})$^72^ from the DFT-calculated free energy of formation of water in the gas phase $({\mu }_{{{H}_{2}O}_{\left(g\right)}}^{{DFT}})$, i.e, ${{\Delta }_{f}G}_{{{H}_{2}O}_{\left(l\right)}}^{0}={{\Delta }_{f}G}_{{{H}_{2}O}_{(g)}}^{{DFT}}-0.09{eV}$.
(iv) Because calculating the energies of dissolved nitrate $({{NO}}_{3\left({aq}\right)}^{-})$ with DFT is problematic^37^, here we use $\frac{1}{2}$ H_2(g)_ and HNO_3(g)_ as references, as shown in Eq. 8:

8 $${HN}{O}_{3(g)}\to {{NO}}_{{3}\,({aq})}^{-}+{H}^{+}$$

We note that HNO_3(g)_ dissociation in Eq. 8 is complete, as it is a strong acid^80^. The free energy of Eq. 8$({\Delta G}_{\,8}^{\exp })$ can be expressed as:

9 $${{\Delta G}}_{8}^{\exp }={\Delta }_{f}G_{{{NO}}_{{3}({aq})}^{-}}^{0}+\frac{1}{2}{{\Delta }}_{f}G_{{H}_{2(g)}}^{0}-{{\Delta }}_{f}G_{{{HNO}}_{3(g)}}^{0}$$

As shown in Fig. 1, ${\Delta G}_{8}^{\exp }$ corresponds to the sum of the solvation and dissociation energies of HNO_3_ $({\Delta }_{{solv}+{diss}}{G}_{{{HNO}}_{3}}^{\exp })$, which can be calculated as the difference between the experimental formation energy of ${{NO}}_{3\left({aq}\right)}^{-}$ (−1.15 eV) and that of HNO_3(g)_ (−0.76 eV)^72^. Hence, in this study ${\Delta G}_{8}^{\exp }={\Delta }_{{solv}+{diss}}{G}_{{{HNO}}_{3}}^{\exp }=-0.39{eV}$. Based on Eq. 9, the free energy of formation of ${{NO}}_{3\left({aq}\right)}^{-}$ can be assessed from DFT and experimental data as:

10 $${{\Delta }_{f}G}_{{{NO}}_{3\left({aq}\right)}^{-}}^{{DFT}}={{\Delta }_{f}G}_{{{HNO}}_{3\left(g\right)}}^{{DFT}}-{\mu }_{{H}^{+}}^{{DFT}}-0.39\,{eV}$$

where the energetics of protons (${\mu }_{{H}^{+}}^{{DFT}}$) is obtained by invoking the computational hydrogen electrode^79^. With these considerations, DFT and experimental values can be combined to semiempirically calculate the free energies of each compound in Eqs. 2 to 7, and the associated free energies of reaction.

### Gas-phase error assessment

An important consideration in the modeling of heterogenous (electro)catalytic reactions is the detection and correction of the errors in the DFT-calculated energies of gas-phase compounds. The errors of O_2(g)_, N_2(g)_, NO_(g)_, HNO_3(g)_, CO_(g)_, and CO_2(g)_, have previously been calculated using several functionals and large values have been reported in various cases^36,38–40,45,53^. These significant errors prevent accurate estimations of important quantities in catalysis for the three reasons detailed below.

First, an accurate equilibrium potential for a given reaction may only be rationally obtained by correcting the gas-phase errors of all reactants and products. This is because the equilibrium potential (*U*_*eq*_) is a function of the reaction free energy (${\Delta }_{r}G$) and the number of electrons transferred ($n$). For a reduction reaction: ${U}_{{eq}}={-\Delta }_{r}G/n.$ For example, the reaction in Eq. 2 has an experimental ${\Delta }_{r}G$ of -0.69 eV and involves 4 proton-electron transfers. Thus, its experimental ${U}_{{eq}}$ is $\frac{-0.69\,{eV}}{-4{e}^{-}}=0.17\,V$. Now, the ${\Delta }_{r}G$ using uncorrected PBE is -1.68 eV and ${U}_{{eq}}$ is $\frac{-1.68\,{eV}}{-4{e}^{-}}=0.42\,V$, which deviates by 0.250 V from the experimental value. When the errors of N_2_ and CO (the reactants) are corrected, the new PBE equilibrium potential is 0.22 V, which is 0.05 V away from the experimental value. After correcting the error in urea, the PBE calculations match the experimental value.

Second, if the potential-limiting step involves molecules, correcting the gas-phase error or not may lead to different qualitative and quantitative conclusions because the reaction energy experiences a shift.

Third, when the overpotential is calculated, gas-phase errors are always important because the overpotential is the difference between a given potential and the one at equilibrium (for a reduction reaction: $\eta ={U}_{{eq}}-U$).

It has also been shown that gas-phase errors can affect adsorption-energy scaling relations and volcano plots^36,39,44^, impairing the predictive capability of descriptor-based models of customary use in computational electrocatalysis.

To obtain a general expression to assess the gas-phase errors, we first consider the formation reaction of a hypothetical compound ${H}_{\alpha }{C}_{\beta }{N}_{\gamma }{O}_{\delta }$:

11 $${\frac{\alpha }{2}H}_{2\left(g\right)}+\beta {C}_{\left(s\right)}+\frac{\gamma }{2}{N}_{2\left(g\right)}+\frac{\delta }{2}{O}_{2\left(g\right)}\to {H}_{\alpha }{C}_{\beta }{N}_{\gamma }{O}_{\delta }$$

where $\alpha ,\,\beta ,\,\gamma ,$ and $\delta$ are integers, the molecules are in their standard states, and ${C}_{\left(s\right)}$ is modeled as graphene. The total error in the DFT-calculated free energy of formation of ${H}_{\alpha }{C}_{\beta }{N}_{\gamma }{O}_{\delta }$, denoted ${\varepsilon }_{T}$, is determined as the difference between the DFT prediction (${\Delta }_{f}{G}_{{H}_{\alpha }{C}_{\beta }{N}_{\gamma }{O}_{\delta }}^{{DFT}}$) and the experimental value $({\Delta }_{f}{G}_{{H}_{\alpha }{C}_{\beta }{N}_{\gamma }{O}_{\delta }}^{\exp })$ as shown in Eq. 12:^36,38–40,44,45,53^

12 $${\varepsilon }_{T}={\Delta }_{f}{G}_{{H}_{\alpha }{C}_{\beta }{N}_{\gamma }{O}_{\delta }}^{{DFT}}-{\Delta }_{f}{G}_{{H}_{\alpha }{C}_{\beta }{N}_{\gamma }{O}_{\delta }}^{\exp }$$

In addition, the total error is the difference between the individual errors of the products and reactants of Eq. 11:^36,38–40,44,45,53^

13 $${\varepsilon }_{T}={\varepsilon }_{{H}_{\alpha }{C}_{\beta }{N}_{\gamma }{O}_{\delta }}-{\frac{\alpha }{2}\varepsilon }_{{H}_{2\left(g\right)}}-{\beta \varepsilon }_{{C}_{\left(s\right)}}-{\frac{\gamma }{2}\varepsilon }_{{N}_{2\left(g\right)}}-{\frac{\delta }{2}\varepsilon }_{{O}_{2\left(g\right)}}$$

The DFT error of N_2(g)_ is calculated from the ammonia synthesis reaction (${\frac{1}{2}N}_{2\left(g\right)}+{\frac{3}{2}H}_{2\left(g\right)}\to {{NH}}_{3\left(g\right)}$)^38^ and ${\varepsilon }_{{O}_{2\left(g\right)}}$ from the water formation reaction (${H}_{2\left(g\right)}+{\frac{1}{2}O}_{2\left(g\right)}\to {H}_{2}{O}_{\left(g\right)}$)^35,79^. Assuming that DFT provides an accurate description of the energetics of H_2(g)_ and C_(s)_ (i.e, ${\varepsilon }_{{H}_{2\left(g\right)}}\approx {\varepsilon }_{{C}_{\left(s\right)}}\approx 0$)^30^, combining Eqs. 12 and 13, and reorganizing, we find an expression for the assessment of the gas-phase error of ${H}_{\alpha }{C}_{\beta }{N}_{\gamma }{O}_{\delta }$, see Eq. 14.

14 $${\varepsilon }_{{H}_{\alpha }{C}_{\beta }{N}_{\gamma }{O}_{\delta \left(g\right)}}=\left({\Delta }_{f}{G}_{{H}_{\alpha }{C}_{\beta }{N}_{\gamma }{O}_{\delta \left(g\right)}}^{{DFT}}+\frac{\gamma }{2}{\varepsilon }_{{N}_{2}}+\frac{\delta }{2}{\varepsilon }_{{O}_{2}}\right)-{\Delta }_{f}{G}_{{H}_{\alpha }{C}_{\beta }{N}_{\gamma }{O}_{\delta }}^{\exp }$$

A detailed example of the use of these equations for urea is presented in Supplementary Note 2. We note that the experimental standard free energies of formation and heats of formation of the molecules under study are reported within chemical accuracy (4.18 kJ mol^−1^ or 0.04 eV). In fact, the errors reported on the NIST website for the heats of formation of CO_2_, CO, water, and urea are 0.13, 0.17, 0.040, and 1.2 kJ mol^−1^ (refs. ^71,81^), respectively, and the errors are smaller in the ATcT database^82^. In addition, the FreeSolv database commonly reports 2.51 kJ mol^−1^ as the uncertainty of the experimental hydration free energies^76^. Hence, the experimental values of interest are known to a greater precision compared to the DFT results, which usually involve errors above 0.1 eV (~10 kJ mol^−1^).

We remark that errors may also exist in the adsorbed state. However, as GGA functionals accurately describe the atomic structure, cohesive energy, and bulk moduli of transition metals and their low Miller-index surfaces^29,31^, we expect these errors to be smaller than those of the gas phase. The error assessment detailed in this section may be extended to adsorbates if accurate experimental adsorption energies are available, but these are scarce in the literature^83^. We are aware of two approaches to estimate errors in the adsorbed state. Based on uncertainty considerations, the first method links gas-phase errors to those of the corresponding adsorbates and provides specific corrections for a given species on a substrate (e.g, *COOH on Cu(111))^43^. Without comparing to experiments, the second method identifies systematic errors of a given adsorbate on a substrate by comparing the adsorption energies using a variety of DFT setups, (i.e, *O on RuO_2_(110))^84^.

To close this section, we remark that including or neglecting the thermal contributions in Eq. 1 may shift the values of the gas-phase errors. These effects are detailed in Supplementary Note 4. Supplementary Table 6 shows the difference of the errors with and without thermal enthalpic contributions from 0 to 298.15 K.

## Results

### Errors in co-electrolysis reactions

**Fig. 2:**
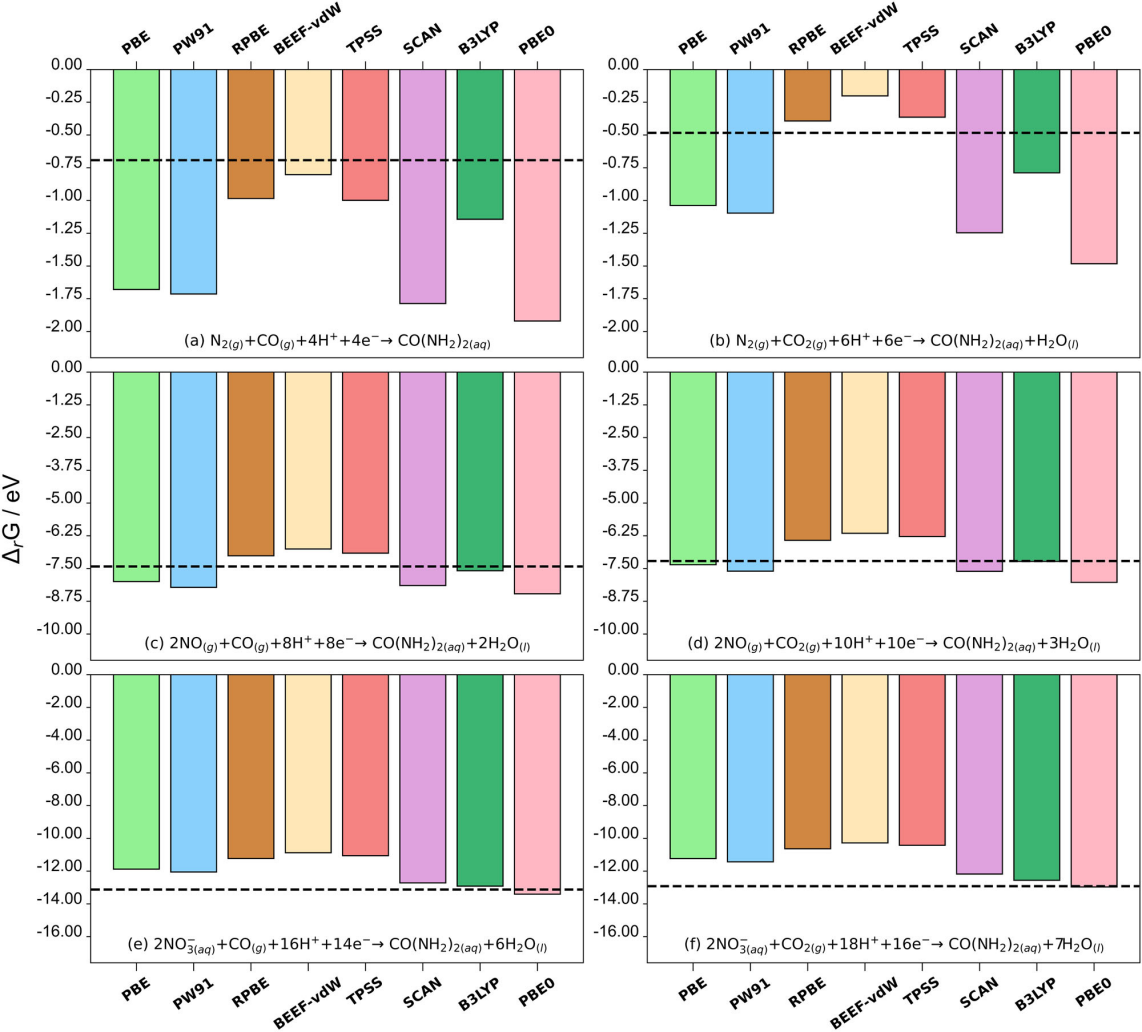
Errors in co-electrolysis reactions. Free energies (${\Delta }_{r}G$) of six co-electrolysis reactions calculated with several exchange-correlation functionals: urea production from the co-electrolysis of (**a**) ${N}_{2\left(g\right)}$ and ${{CO}}_{\left(g\right)}$, (**b**) ${N}_{2\left(g\right)}$ and ${{CO}}_{2\left(g\right)}$, (**c**) ${{NO}}_{\left(g\right)}$ and ${{CO}}_{\left(g\right)}$, (**d**) ${{NO}}_{\left(g\right)}$ and ${{CO}}_{2\left(g\right)}$, (**e**) ${{NO}}_{3({aq})}^{-}$ and + ${{CO}}_{\left(g\right)}$, (**f**) ${{NO}}_{3({aq})}^{-}$ and ${{CO}}_{2\left(g\right)}$. In all panels, the respective experimental energy is shown as a dashed black line. The DFT energies of the molecules do not include any gas-phase corrections, see the values of these corrections in Table 1.

The experimental and DFT-calculated energies of the studied reactions are shown in Supplementary Table 4 and Fig. 2. These values were obtained using the uncorrected DFT energies in Supplementary Table 3 as explained in Supplementary Note 3. For each functional used, the mean absolute errors (xc-MAEs) and maximum absolute errors (xc-MAXs) with respect to experiments are shown in Supplementary Table 4. In addition, Supplementary Table 4 contains the mean and maximum absolute errors for each reaction (r-MAE and r-MAX, respectively).

The r-MAEs and r-MAXs in Supplementary Table 4 indicate that, for the six co-electrolysis reactions studied here, pure GGA-based DFT calculations yield significant deviations with respect to experiments, with r-MAEs spanning from 0.47 to 1.47 eV, and r-MAX values in the range of 1.00 to 2.64 eV. Note in passing that the reaction energies, r-MAE and r-MAX tend to increase as the reactants are more oxidized, as shown in Supplementary Fig. 2. Moreover, the large mean and maximum absolute errors for each functional in Supplementary Table 4 (xc-MAE and xc-MAX), indicate that none of the studied functionals correctly describes all the co-electrolysis reactions, regardless of the functional rung on Jacob’s ladder. In fact, for GGA functionals the average xc-MAE and xc-MAX are 0.97 and 2.02 eV; for meta-GGA functionals they are 0.88 and 1.80 eV; and for the hybrid functionals they are 0.49 and 0.84 eV, respectively. Hence, there is an error decrease upon climbing Jacob’s ladder, but even hybrid functionals display considerable deviations.

The panels in Fig. 2 aid in visualizing the large discrepancies between theory and experiments. In all the reactions, most of the bars lie far from the experimental value (dashed line in Fig. 2). Consistent with previous works, PBE and PW91 display comparable errors for all reactions^85,86^. Interestingly, BEEF-vdW, RPBE, and TPSS display similar reaction energies in all panels of Fig. 2. In contrast, SCAN and TPSS present large differences although both are meta-GGA functionals. Some similarities are observed in panels e and f for the hybrids, where PBE0 performs better than B3LYP, but significant differences are observed as the N-containing reactant becomes less oxidized (panels a–d) and the PBE0 accuracy worsens with respect to B3LYP. As expected, the two hybrid functionals provide more accurate values than GGA and meta-GGA functionals. Overall, calculations using B3LYP lead to results with the smallest errors, presumably as a consequence of its parameterization based on thermochemical data such as atomization energies and ionization potentials. However, for some reactions, the errors of the hybrid functionals surpass the accuracy necessary to allow for accurate predictions ($$ < 0.1{eV}$$): B3LYP yields errors of −0.45, −0.31, and 0.36 eV for reactions a, b, and f, and PBE0 yields errors of −1.23, −1.00, −1.04, −0.81, and −0.28 eV for the reactions in Fig. 2a–e.

### Errors in the molecules

Table 1 summarizes the gas-phase errors of the species involved in the co-electrolysis reactions for all the functionals under analysis using Eq. 14. Note that ${{NO}}_{3\left({aq}\right)}^{-}$ and CO(NH_2_)_2(aq)_ display the same errors as their respective gaseous references, HNO_3(g)_ and CO(NH_2_)_2(g)_. This is because the energies of these species were calculated semiempirically from DFT energies of the gases and experimental values (see section 2.2).

**Table 1:** Individual gas-phase errors.

Species	PBE	PW91	RPBE	BEEF-vdW	TPSS	SCAN	B3LYP	PBE0
N_2(g)_	0.49	0.52	0.11	−0.16	0.00	0.47	0.28	0.71
O_2(g)_	−0.42	−0.27	−0.70	−0.78	−0.80	−0.40	−0.28	−0.17
NO_(g)_	0.04	0.15	−0.29	−0.47	−0.41	0.05	0.00	0.26
CO_(g)_	0.30	0.30	−0.04	−0.13	−0.05	0.30	−0.03	0.40
CO_2(g)_	−0.14	−0.11	−0.42	−0.52	−0.47	−0.03	−0.18	0.18
HNO_3(g)_	−0.88	−0.79	−1.04	−1.26	−1.19	−0.51	−0.19	−0.12
${{{{{{\rm{NO}}}}}}}_{3\left({{{{{\rm{aq}}}}}}\right)}^{-}$	−0.88	−0.79	−1.04	−1.26	−1.19	−0.51	−0.19	−0.12
CO(NH_2_)_2(g)_	−0.20	−0.20	−0.22	−0.40	−0.36	−0.32	−0.20	−0.11
CO(NH_2_)_2(aq)_	−0.20	−0.20	−0.22	−0.40	−0.36	−0.32	−0.20	−0.11
MAE	0.39	0.37	0.45	0.60	0.54	0.32	0.17	0.24
MAX	0.88	0.79	1.04	1.26	1.19	0.52	0.28	0.71

DFT errors in the formation energy of the species involved in the co-electrolysis reactions. The MAEs and MAXs are reported for each functional. All values are in eV.

As shown in Table 1, the DFT errors of most species under study are significant regardless of the functional rung on Jacob’s ladder, and reach in some cases values more negative than −1 eV. This is the case of HNO_3(g)_ using RPBE (−1.04 eV), BEEF-vdW (−1.26 eV), and TPSS (−1.19 eV). The hybrid functionals B3LYP and PBE0 yield the lowest gas-phase errors (MAEs of 0.17 and 0.24 eV in Table 1) but still exhibit large MAX figures (0.28 and 0.71 eV). In fact, PBE0 displays the largest error for N_2(g)_ (0.71 eV), which partly explains the substantial deviations of this functional in Fig. 2a, b.

The values in Table 1 can be employed to rapidly estimate the error cancellation of a functional when modeling a chemical reaction. Error cancellation may lead to accurate predictions of reaction energies. For instance, in Fig. 2f we observe for PBE0 an almost complete error cancellation because the errors of reactants and products differ only by 0.05 eV: ${\varepsilon }_{{urea}}-2\cdot {\varepsilon }_{{{NO}}_{3\left({aq}\right)}^{-}}-{\varepsilon }_{{{CO}}_{2}}=-0.11{eV}-2\cdot \left(-0.12{eV}\right)-\left(0.18{eV}\right)\approx -0.05{eV}$. Furthermore, the errors in N_2(g)_ and O_2(g)_ are large for all functionals, spanning from -0.16 to 0.71 eV for N_2(g),_ and from -0.80 to -0.17 eV for O_2(g)_. For urea, significant errors are found for all scrutinized functionals with PBE0 presenting the lowest value (-0.11 eV) and, surprisingly, BEEF-vdW displaying the largest (-0.40 eV). It is worth noting that this range of errors for urea is narrow compared to the other molecules in Table 1 and that all values are negative. Hence, a tentative estimate for the DFT-based error for the formation energy of urea is -0.25 ± 0.10 eV, which corresponds to the average and standard deviation of the corresponding values in Table 1.

**Fig. 3:**
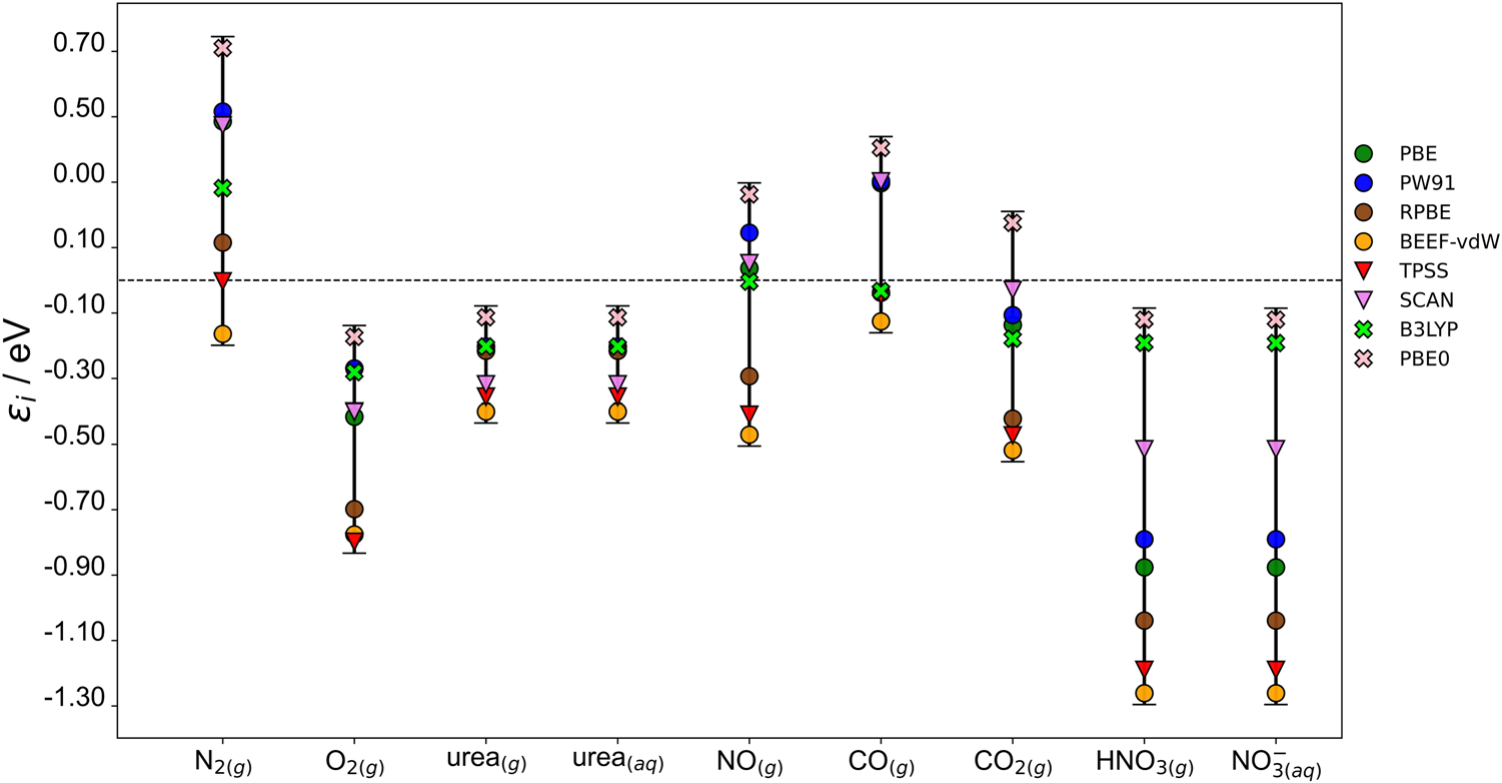
Individual errors of the species. DFT errors (${\varepsilon }_{i}$) for the compounds involved in the co-electrolysis reactions. Circles (●) are for GGA errors, triangles (▼) are for meta-GGA errors, and crosses (**×**) are for hybrid errors. The vertical bars correspond to the ranges spanned by the functionals. All values are in eV.

Figure 3 provides a graphical representation of the values in Table 1. The ranges of the errors are larger than 0.20 eV in all cases. For most molecules, we observe that RPBE, BEEF-vdW, and TPSS exhibit the largest negative errors. In contrast, PBE0 always yields the largest positive deviations for N_2(g)_ and CO_(g)_ but also the lowest errors for O_2(g)_, urea and HNO_3(g)_/NO^-^_3(aq)_. Finally, while extent of the error fluctuation of PBE, PW91, and SCAN over the whole set of molecules is rather similar, B3LYP shows the smallest and relatively stable set of errors, all relatively close to zero. However, as mentioned before, our general conclusion is that none of the functionals in Table 1 yields satisfactory energies for the co-electrolysis reactions under analysis.

Importantly, if experimental results are not available to calculate the gas-phase errors using Eq. 14, one could rely on highly accurate quantum chemical methods based on wave-function theory such as CCSD(T) using large basis sets. Alternatively, one can use correction approaches based on structural features, such as the number of oxygen atoms in the molecule^39^, the presence of certain functional groups^38^, or the occurrence of specific chemical structures within the compound, such as CO-, OCO-, ONO-, NNO-, or -NOH backbones^41,43,53^. For instance, from a functional group perspective, CO(NH_2_)_2(g)_ can be considered an amide with an amino group bound to it. The respective PBE errors of the amino and amide groups are 0.00 and −0.17 eV^38^, yielding a total error of −0.17 eV, which agrees well with that in Table 1 (−0.20 eV). This approximation is somewhat satisfactory for the other GGAs studied (PW91: −0.15 eV, RPBE: −0.16 eV, BEEF-vdW: −0.47 eV^38^ vs −0.20, −0.22 and −0.40 eV in this study). However, we note that ${\varepsilon }_{{{{CO}}}({NH}_{2})_{2\left(g\right)}}\approx {\varepsilon }_{{amide}\left(g\right)}+\frac{1}{2}{\varepsilon }_{{amine}\left(g\right)}$ yields a more accurate approximation (PBE: −0.17 eV, PW91: −0.15 eV, RPBE: −0.18 eV, BEEF-vdW: −0.42 eV)^38^. This is because of the double counting of one of the C-N bonds: the amide correction was designed to account for the error in the bond between an sp^3^ C and -CO(NH_2_), and the amine correction for the bond between an sp^3^ C and -NH_2_.

### Implications for electrocatalysis

**Fig. 4:**
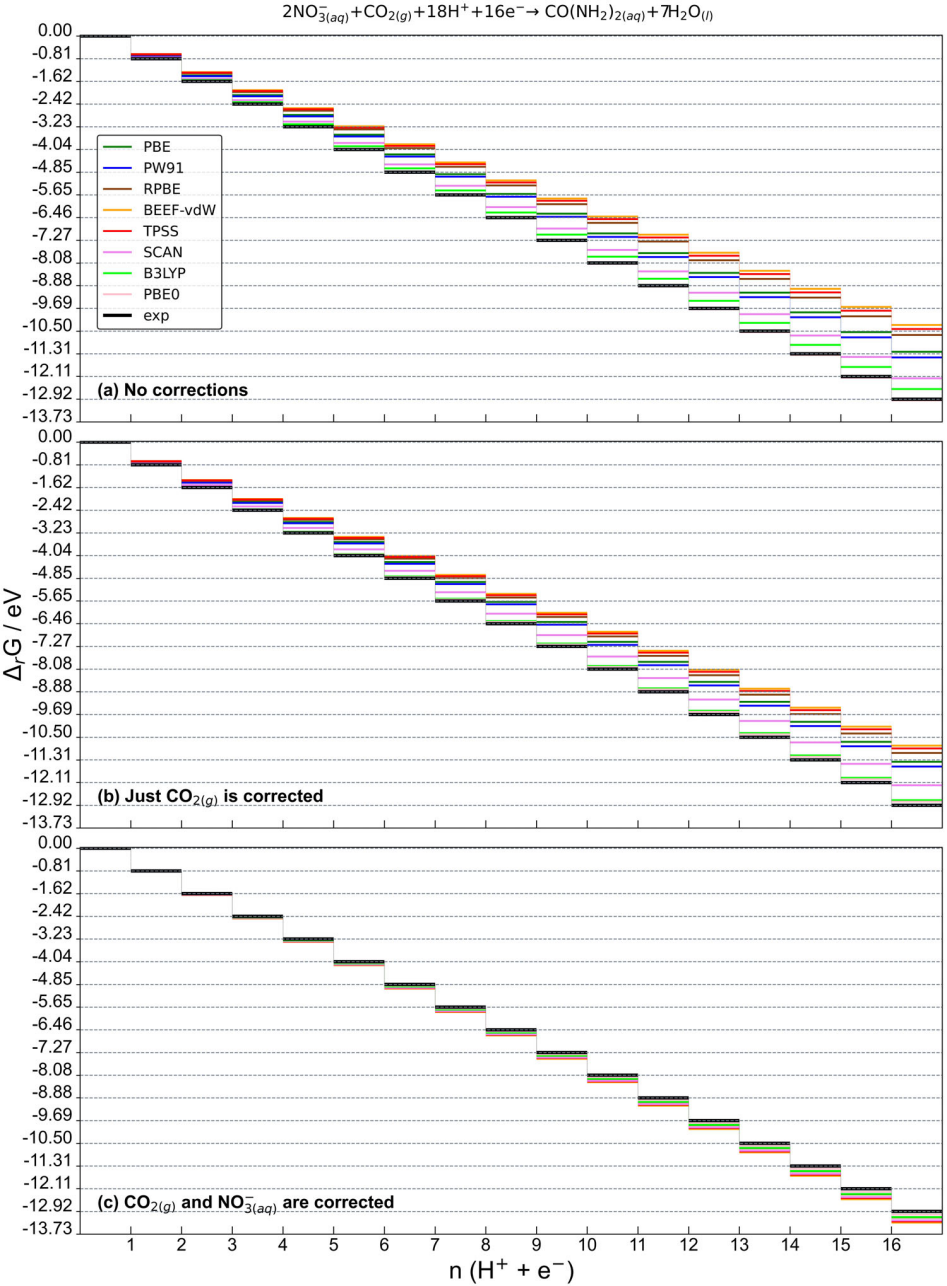
Free energy diagrams for the co-electrolysis of $N{O}_{3(aq)}^{-}$ and CO2(g) to urea on the ideal catalyst. The diagrams were built using (**a**) uncorrected DFT energies (Supplementary Table 4), (**b**) the uncorrected DFT energies of ${{NO}}_{3\left({aq}\right)}^{-}$ and CO(NH_2_)_2(aq)_, and the corrected energy of CO_2(g)_, (**c**) the corrected energies of ${{NO}}_{3\left({aq}\right)}^{-}$ and CO_2(g)_, and the uncorrected DFT energy of CO(NH_2_)_2(aq)_. Ideal values from experiments are in black. The *y*-axis scale is divided into multiples of the experimental equilibrium potential.

To illustrate the effect of gas-phase errors on electrocatalysis, the DFT-uncorrected values in Supplementary Table 4 and the errors in Table 1 were used to build free-energy diagrams of the thermodynamically ideal catalyst for each co-electrolysis reaction, see Fig. 4 and Supplementary Figures 3-7 in the Supplementary Note 5. The concept of an ideal catalyst is commonly employed in electrocatalysis to outline the most efficient conversion that conforms to the first and second laws of thermodynamics^87–89^, thus serving as a benchmark for real catalysts. In an ideal catalyst, the reaction energy of all electrochemical steps is the same and corresponds to the overall reaction energy divided by the number of electrons transferred (i.e, $\Delta {G}_{i}={\Delta }_{r}G/n$). Numerically, the magnitude of the ideal electrochemical steps is identical to the equilibrium potential. Hence, the chemical identity of the intermediates need not be known to build the ideal free-energy diagram. In contrast, real catalysts usually display asymmetric free-energy diagrams and require knowledge of the chemical identity and energetics of the intermediates. For the co-reduction of ${{NO}}_{3\left({aq}\right)}^{-}$ and CO_2(g)_ in Fig. 4, previous works proposed the following mechanism:^20,90,91^ *NO_3_ reduction to *NO_2_, then coupling with *CO_2_ to form *CO_2_NO_2_. Subsequent protonation of *CO_2_NO_2_ yields *CO_2_NH_2_, which in turn reduces to *COOHNH_2_ in the potential-determining step^92^. *COOHNH_2_ reduces to *CONH_2_, which couples to *NO_2_, producing *CONO_2_NH_2_. Finally, *CONO_2_NH_2_ is hydrogenated twice to give urea.

Figure 4 shows three energy diagrams of the ${{NO}}_{3\left({aq}\right)}^{-}$ and CO_2(g)_ co-electrolysis reaction to urea on the ideal catalyst (Eq. 7). Panel a was built with the uncorrected DFT values, i.e, with no gas-phase corrections. In panel b, the error of CO_2(g)_ (the C-containing reactant) was accounted for, leaving the DFT energies of both ${{NO}}_{3\left({aq}\right)}^{-}$ and CO(NH_2_)_2(aq)_ uncorrected. In panel c, the DFT-energies of CO_2(g)_and ${{NO}}_{3\left({aq}\right)}^{-}$ were corrected, while the energy of CO(NH_2_)_2(aq)_ remained uncorrected, except for the black line, in which DFT and experiments coincide.

Figure 4a shows that all functionals diverge from the free-energy profile of the ideal catalysts (calculated on the basis of experimental values) as more electrochemical steps are considered, reaching a maximum deviation at the last step of the catalytic pathway. This maximum deviation corresponds to the difference between the DFT reaction energy and its experimental counterpart. The telescopic effect in Fig. 4a also occurs in panels b and c, but with nuances introduced by the partial corrections. We note the total error can also be obtained by assessing the difference between the errors of reactants and products. For example, based on the values in Table 1, for Eq. 7 and BEEF-vdW the resulting error is ${2\cdot \varepsilon }_{{{NO}}_{3\left({aq}\right)}^{-}}+{\varepsilon }_{{{CO}}_{2(g)}}-{\varepsilon }_{{{{CO}}}({NH}_{2})_{2\left({aq}\right)}}=2\cdot -1.26-0.52+0.40=-2.64{eV}$. In Fig. 4c the departures of the predicted values from calculations using the various functionals with respect to those from experiments stem from the error in CO(NH_2_)_2(aq)_, as it is the only remaining uncorrected species. In other words, the difference between DFT-based and experimental values for the last reaction step of Fig. 4c is ${\varepsilon }_{{{{CO}}({NH}_{2})}_{2\left({aq}\right)}}$. Moreover, we note that after correcting the error of urea using the respective values in Table 1, all the gas-phase errors are accounted for and the “DFT + corrections” diagram becomes that of the ideal catalyst, which is shown in black in all three panels of Fig. 4.

Analogous diagrams for the other reactions under study are given in Supplementary Figs. 3–7. We emphasize that the conclusions drawn from Fig. 4 also hold for these Supplementary figures. Since ${\varepsilon }_{{{{CO}}({NH}_{2})}_{2\left({aq}\right)}}$ < 0 for all the functionals assessed (see Table 1), the DFT-calculated lines are always below the experimental values in the bottom panels of Fig. 4 and Supplementary Figs. 3–7.

## Conclusions

Simultaneous electrocatalytic reduction of nitrogen and carbon pollutants to produce urea is an appealing alternative to help remediate the colossal imbalances of the nitrogen and carbon cycles. Herein, we showed how experimental data can be coupled with DFT-calculated gas-phase energies to model six co-reduction reactions of carbon and nitrogen oxides to produce hydrated urea. The average MAE/MAX values versus experiments are 0.97 eV/2.02 eV for GGA functionals (PBE, PW91, RPBE, and BEEF-vdW), while those of meta-GGAs (TPSS and SCAN) are 0.88 eV/1.80 eV, and those of the hybrids (PBE0, B3LYP) are 0.49 eV/0.84 eV. Hence, the use of DFT to model these reactions entails large errors, even for hybrid functionals, indicating that accurate predictions are only attained once the DFT errors of all the molecules under study are corrected.

Moreover, the DFT error in the formation energy of urea spans a relatively narrow range of values, such that -0.25 ± 0.10 eV is a reasonable error estimate for DFT calculations, although the use of specific corrections is always more advisable than an average.

The effect of these numerical deviations in catalysis was illustrated for the free-energy diagrams of the ideal electrocatalyst extracted from experimental data for various co-electrolysis reactions. The departures of DFT predictions from the experimental trends are substantial for all functionals. However, we showed that the errors can easily be corrected in a semiempirical manner.

All this hints toward the need for an assessment of gas-phase errors at the early stages of computational electrocatalysis research to avoid potentially inaccurate and misleading conclusions, regardless of the chosen rung on Jacob’s ladder of density functional approximations.

### Supplementary information

Supplemental Information

## Data Availability

The authors declare that data supporting the findings of this study are available within the paper and its supplemental material file. Additional data are available from the corresponding author upon reasonable request.
