# Supplementary material for: Minimum conditions for accurate modeling of urea production via co-electrolysis
Source: Commun Chem. 2023 Sep 13;6:196. doi: 10.1038/s42004-023-00990-7 (PMC10499819; doi:10.1038/s42004-023-00990-7)
Supplement: Supplementary file 1 — Supplemental Information [file 42004_2023_990_MOESM1_ESM.pdf]

Supplemental Material

Minimum Conditions for Accurate Modelling of Urea Production *via* Co-electrolysis

Ricardo Urrego-Ortiz,[1,2] Santiago Builes,[3] Francesc Illas,[2] Stefan T. Bromley,[2] Marta Costa Figueiredo,[4] Federico Calle-Vallejo[2,5,*]

[1] *Departament de Ciència de Materials i Química Física & Institut de Química Teòrica i Computacional (IQTCUB), Universitat de Barcelona,C/ Martí i Franquès 1, 08028 Barcelona, Spain.*

[2] *Nano-Bio Spectroscopy Group and European Theoretical Spectroscopy Facility (ETSF), Department of Polymers and Advanced Materials: Physics, Chemistry and Technology, University of the Basque Country UPV/EHU, Av. Tolosa 72, 20018 San Sebastián, Spain.*

[3] *Escuela de Ciencias Aplicadas e Ingeniería, Universidad EAFIT, Carrera 49 # 7 sur 50, 050022, Medellín, Colombia.*

[4] *Eindhoven Institute of Renewable Energy Systems (EIRES). Eindhoven University of Technology, PO Box 513, Eindhoven 5600 MB, The Netherlands.*

[5] *IKERBASQUE, Basque Foundation for Science, Plaza de Euskadi 5, 48009 Bilbao, Spain.*

*Correspondence to: federico.calle@ehu.es

Table of contents

Supplementary note 1: Convergence tests **2**

Supplementary note 2: Pinpointing and correcting gaseous errors **3**

Supplementary note 3: DFT-calculated free energies of reaction **6**

Supplementary note 4: Thermal contributions **9**

Supplementary note 5: Free-energy diagrams **10**

Supplementary note 6: Alternative thermodynamic cycles **16**

Supplementary note 7: Calculation settings **16**

Supplementary note 8: Direct coordinates **17**

Supplementary references **18**

Supplementary note 1: Convergence tests

To assure converged DFT energies and ZPEs, we calculated $\Delta \boldsymbol{E}^{\boldsymbol{DFT}}$ of the gaseous urea production from $CO_{2(g)}$ and $N_{2(g)}$ ($\boldsymbol{N_{2(g)}} + \boldsymbol{CO_{2(g)}} + \boldsymbol{3H_{2(g)}} \rightarrow \boldsymbol{CO(NH_2)_{2(g)}} + \boldsymbol{H_2O_{(g)}}$) using the PBE exchange-correlation functional and several energy cutoffs from 400 to 1000 eV. Supplementary Figure 1 shows the $\Delta_r \boldsymbol{E}^{\boldsymbol{DFT}}$ calculated for each cutoff. Moreover, the $\Delta_r \boldsymbol{ZPE}$ for this reaction was calculated using an energy cutoff of 450 and 1000 eV. The values are shown in Supplementary Table 1. From both Supplementary Figure 1 and Supplementary Table 1, working with a plane-wave cutoff of 450 eV guarantees converged DFT reaction energies and Δ_rZPEs below 0.05 eV.

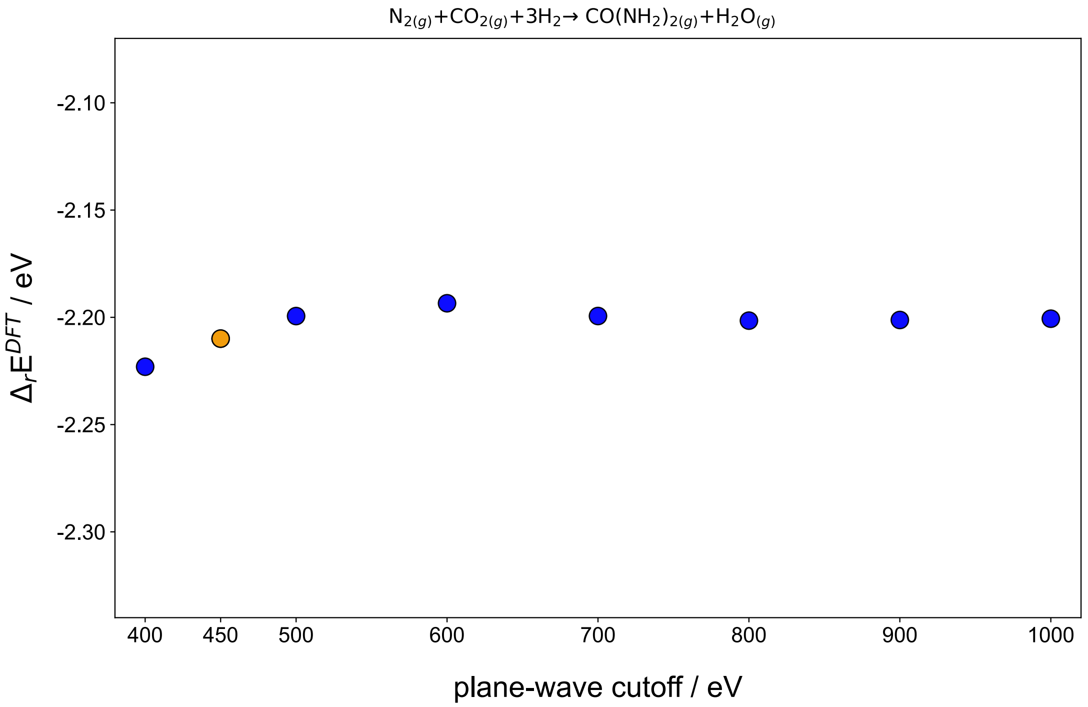

Supplementary Figure 1. $\Delta_r \boldsymbol{E}^{\boldsymbol{DFT}}$ for the co-reduction of $N_{2(g)}$ and $CO_{2(g)}$ to gaseous urea calculated at different energy cutoffs. In orange, the cutoff of 450 eV used in this work.

Supplementary Table 1. ZPEs for the compounds involved in the gaseous urea production and the corresponding ΔZPE calculated within the harmonic approximation with energy cutoffs of 450 and 1000 eV.

cutoff	$N_{2(g)}$	$CO_{2(g)}$	$H_{2(g)}$	$CO(NH_2)_{2(g)}$	$H_2O_{(g)}$	ΔZPE
450	0.15	0.31	0.28	1.69	0.58	0.96
1000	0.15	0.31	0.28	1.69	0.58	0.97

Supplementary note 2: Pinpointing and correcting gaseous errors

In this section, we show in a step-by-step fashion how the error in the DFT energy of gaseous urea ($\boldsymbol{CO(NH_2)_{2(g)}}$) is determined. The values of the SCAN xc-functional are used as a case study.

Step 1: difference between DFT-calculated formation energies and experiments

The formation of $\boldsymbol{CO(NH_2)_{2(g)}}$ from the elements that compose it in their standard states is:

$$2H_{2(g)} + C_{(s)} + N_{2(g)} + \frac{1}{2}O_{2(g)} \rightarrow CO(NH_2)_{2(g)} \quad \text{(S1)}$$

The experimental formation energy of gaseous urea ($\Delta_f \boldsymbol{G^{exp}_{CO(NH_2)_{2(g)}}}$) found in thermodynamic tables is -1.57 eV.[1] On the other hand, the DFT-calculated formation energy of urea ($\Delta_f \boldsymbol{G^{DFT}_{CO(NH_2)_{2(g)}}}$) can be estimated as follows:

$$\Delta_f \boldsymbol{G^{DFT}_{CO(NH_2)_{2(g)}}} = \boldsymbol{G^{DFT}_{CO(NH_2)_{2(g)}}} - \boldsymbol{2G^{DFT}_{H_{2(g)}}} - \boldsymbol{G^{DFT}_{C_{(s)}}} - \boldsymbol{G^{DFT}_{N_{2(g)}}} - \boldsymbol{\frac{1}{2}G^{DFT}_{O_{2(g)}}} \quad \text{(S2)}$$

where $G_i^{DFT} \approx E_i^{DFT} + ZPE_i + (\Delta_f H_{i,@298.15\,K} - \Delta_f H_{i,@0\,K}) - TS_i$ (Equation 1 in the main text) and the energy of graphene is per C atom. The effect of neglecting the enthalpic contributions on the calculation of the errors is analyzed in section S4. The values required to be plugged into Equation S2 are presented in Supplementary Table 2 along with the values of $NH_{3(g)}$ and $H_2O_{(g)}$ which are used in a subsequent analysis.

Supplementary Table 2. DFT-calculated energies (E^{DFT}) and ZPEs, experimental TS, and the differences between the experimental formation enthalpies between 298.15 and 0 K for several gaseous species and for graphene as a proxy of graphite.[2,3] The DFT values are obtained using the SCAN exchange-correlation functional. All values are in eV.

species	E^{DFT}	ZPE	TS	$\Delta_f H_{i\,@298.15\,K} - \Delta_f H_{i\,@0\,K}$
$C_{(s)}$	-10.08	0.13	0.02	0.01
$H_{2(g)}$	-6.90	0.29	0.40	0
$O_{2(g)}$	-12.31	0.11	0.63	0
$N_{2(g)}$	-18.54	0.16	0.59	0
$CO(NH_2)_{2(g)}$	-52.26	1.75	0.86	-0.17
$NH_{3(g)}$	-20.70	0.95	0.60	-0.07
$H_2O_{(g)}$	-15.59	0.59	0.58	-0.03

From Equation 12 in the main text, the total error in the formation reaction of gaseous urea (ε_T) can be calculated as the difference between the DFT and experimental free energies of formation.

$$\varepsilon_T = \Delta_f G^{DFT}_{CO(NH_2)_{2(g)}} - \Delta_f G^{exp}_{CO(NH_2)_{2(g)}} \tag{S3}$$

Step 2: deconvoluting the contributions to the total error

$\boldsymbol{\varepsilon_T}$ for urea is an assortment of the errors in the reactants of Equation S1 and the error in the urea itself. To decouple these contributions, Equation 13 in the main text is used:

$$\boldsymbol{\varepsilon_T = \varepsilon_{CO(NH_2)_{2(g)}} - 2\varepsilon_{H_{2(g)}} - \varepsilon_{C_{(s)}} - \varepsilon_{N_{2(g)}} - \frac{1}{2}\varepsilon_{O_{2(g)}}} \tag{S4}$$

where $\boldsymbol{\varepsilon_T}$ was previously calculated using Equation S3 (or Equation 12 in the main text). At this point, Equation S4 can be simplified in view of the accurate description of the energetics of $H_{2(g)}$ and graphene within standard DFT ($\boldsymbol{\varepsilon_{H_{2(g)}} \approx \varepsilon_{C_{(s)}} \approx 0}$).[4] Thus, the unknowns in Equation S5 are the terms at the right hand side, i.e. the error in urea and those of the diatomic molecules O_2 and N_2, which are typically large and functional-dependent.[5–8]

$$\boldsymbol{\varepsilon_T = \varepsilon_{CO(NH_2)_{2(g)}} - \varepsilon_{N_{2(g)}} - \frac{1}{2}\varepsilon_{O_{2(g)}}} \tag{S5}$$

Step 3: calculating $\boldsymbol{\varepsilon_{N_{2(g)}}}$ and $\boldsymbol{\varepsilon_{O_{2(g)}}}$

The errors in the DFT energies of molecular nitrogen and oxygen can be calculated by applying Equations S2 to S5 to the ammonia synthesis reaction (ASR: $\frac{1}{2}N_{2(g)} + \frac{3}{2}H_{2(g)} \rightarrow NH_{3(g)}$) and water formation reaction (WFR: $H_{2(g)} + \frac{1}{2}O_{2(g)} \rightarrow H_2O_{(g)}$), respectively. From the ASR, Equation S6 is obtained. Analogously, WFR yields Equation S7.

$$\varepsilon_{N_{2(g)}} = -2\,(\Delta_f G_{ASR}^{DFT} - \Delta_f G_{ASR}^{exp}) \quad \text{(S6)}$$

$$\varepsilon_{O_{2(g)}} = -2\,(\Delta_f G_{WFR}^{DFT} - \Delta_f G_{WFR}^{exp}) \quad \text{(S7)}$$

We note that Equations S6 and S7 were simplified by assuming an accurate DFT description of the energetics of $H_{2(g)}$, $H_2O_{(g)}$ and $NH_{3(g)}$ ($\varepsilon_{H_{2(g)}} \approx \varepsilon_{NH_{3(g)}} \approx \varepsilon_{H_2O_{(g)}} \approx 0$). This is in general a good approximation given that $H_2O_{(g)}$ and $NH_{3(g)}$ contain only sigma bonds.[4]

From experiments, $\Delta_f G_{ASR}^{exp} = -0.17\ eV$ and $\Delta_f G_{WFR}^{exp} = -2.37\ eV$.[9] From the values in Supplementary Table 2, $\Delta_f G_{ASR}^{DFT} = -0.41\ eV$ and $\Delta_f G_{WFR}^{DFT} = -2.17\ eV$. Hence, using Equations S6 and S7, $\varepsilon_{N_{2(g)}} = 0.47\ eV$ and $\varepsilon_{O_{2(g)}} = -0.40\ eV$, which are the SCAN values reported in Table 1 of the main text.

Step 4: isolating $\boldsymbol{\varepsilon_{CO(NH_2)_{2(g)}}}$

Combining Equation S3 and Equation S5 leads to Equation 14 in the main text. This equation allows for the calculation of the error of gaseous urea once the errors of the diatomic molecules are known, as shown in Equation S8.

$$\varepsilon_{CO(NH_2)_{2(g)}} = \left(\Delta_f G_{CO(NH_2)_{2(g)}}^{DFT} - \Delta_f G_{CO(NH_2)_{2(g)}}^{exp}\right) + \varepsilon_{N_{2(g)}} + \frac{1}{2}\varepsilon_{O_{2(g)}} \quad \text{(S8)}$$

Note that the total error ε_T can be used in Equation S8 instead of the energy difference. Replacing the known values in Equation S8, namely $\Delta_f G_{CO(NH_2)_{2(g)}}^{DFT} = -2.16\ eV$, $\Delta_f G_{CO(NH_2)_{2(g)}}^{exp} = -1.57\ eV, \varepsilon_{N_{2(g)}} = 0.47\ eV$, $\varepsilon_{O_{2(g)}} = -0.40\ eV$ results in $\varepsilon_{CO(NH_2)_{2(g)}} = -0.32\ eV$, which is the value shown in Table 1 of the main text.

These four steps were followed for all the molecules under study to obtain the values in Table 1. A general trend can be noticed: the more oxidized the compounds, the larger their errors, see Supplementary Figure 2. This is a consequence of DFT's inability to suitably describe multiple bonds,[4] which increasingly form as more oxygen atoms take part in the molecules. This is in line with previous studies performed on oxidized carbon and nitrogen species.[10,11]

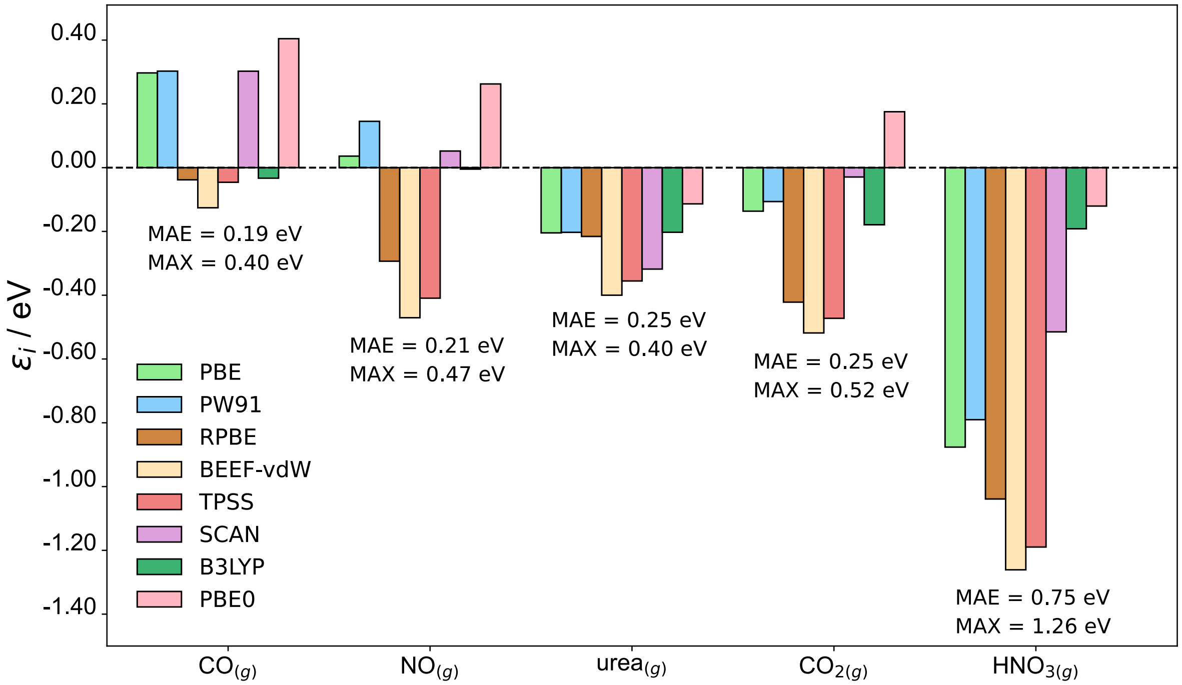

Supplementary Figure 2. Errors in the oxygen-containing compounds studied in this work for various exchange-correlation functionals. The errors increase alongside the number of oxygen atoms within each compound (1 in CO, NO and urea, 2 in carbon dioxide, 3 in nitric acid). Mean and maximum absolute errors are provided for each molecule.

Supplementary note 3: DFT-calculated free energies of reaction

The DFT-calculated free energy of a generic reaction can be estimated by means of Equation S9:

$$\Delta_r G^{DFT} = \sum_i v_i \, G_i^{DFT} = \sum_i v_i \, \Delta_f G_i^{DFT} \tag{S9}$$

where v_i is the stoichiometric number of species i (which is a positive integer for products and a negative integer for reactants), $G_i^{DFT} \approx E_i^{DFT} + ZPE_i + (\Delta_f H_{i,@298.15\,K} - \Delta_f H_{i,@0\,K}) - TS_i$ (Equation 1 in the main text), and $\Delta_f G_i^{DFT}$ is the formation energy of the species i. Note in passing

that in Equation S9 the DFT errors are not accounted for. If these errors are included, the DFT-calculated energy comes closer or even matches the experimental reaction energy depending on the method used to estimate the DFT errors.

Supplementary Table 3 contains the uncorrected DFT formation energies ($\Delta_f \boldsymbol{G_i^{DFT}}$) and the ZPEs of the compounds involved in the co-electrolysis reactions in Equations 2 to 7 in the main text. These energies are shown for all the xc-functionals studied in this work. Supplementary Table 4 shows the uncorrected DFT free energies of reactions in Equations 2 to 7 and includes the mean and maximum absolute errors for each functional (xc-MAE and xc-MAX) and reaction (r-MAE and r-MAX). Supplementary Table 4 and Figure 2 in the main text were built using the uncorrected values in Supplementary Table 3.

Supplementary Table 3. Uncorrected free energies of formation calculated with DFT ($\Delta_f G_i^{DFT}$) and ZPEs (in parenthesis) for the compounds involved in the co-reduction reactions studied in this work (Equations 2 to 7 in the main text) using eight xc-functionals at different levels of theory.

species	exp	PBE	PW91	RPBE	BEEF-vdW	TPSS	SCAN	B3LYP	PBE0
$NO_{(g)}$	0.91	0.91 (0.12)	0.93 (0.12)	0.91 (0.12)	0.91 (0.12)	0.90 (0.13)	0.92 (0.13)	0.90 (0.14)	0.90 (0.14)
$NO^{-}_{3(aq)}$[a]	-1.15	-1.65 (0.70)	-1.80 (0.70)	-1.20 (0.69)	-1.17 (0.70)	-1.14 (0.69)	-1.30 (0.72)	-1.06 (0.72)	-1.37 (0.77)
$CO_{(g)}$	-1.42	-0.92 (0.13)	-0.98 (0.13)	-1.11 (0.13)	-1.16 (0.14)	-1.07 (0.13)	-0.92 (0.14)	-1.31 (0.14)	-0.93 (0.15)
$CO_{2(g)}$	-4.09	-3.81 (0.31)	-3.92 (0.31)	-3.81 (0.31)	-3.83 (0.31)	-3.76 (0.32)	-3.72 (0.33)	-3.99 (0.32)	-3.74 (0.34)
$H_2O_{(l)}$[a]	-2.46	-2.25 (0.57)	-2.32 (0.58)	-2.11 (0.58)	-2.07 (0.59)	-2.06 (0.57)	-2.26 (0.59)	-2.32 (0.60)	-2.37 (0.63)
$CO(NH_2)_{2(aq)}$[a]	-2.11	-2.60 (1.69)	-2.70 (1.69)	-2.10 (1.69)	-1.96 (1.71)	-2.07 (1.70)	-2.71 (1.75)	-2.46 (1.74)	-2.85 (1.80)

[a]The ZPEs of the aqueous and liquid species correspond to the ZPEs of the gas-phase reference

Supplementary Table 4. Experimental and DFT-calculated free energies of various co-electrolysis reactions that produce urea using several xc functionals. The mean absolute errors (MAE) and maximum absolute errors (MAX) are shown for each xc-functional and are referred to as xc-MAE and xc-MAX, respectively. On the other hand, the MAE and MAX values for each reaction are also presented (r-MAE and r-MAX, respectively). All values are in eV.

reaction	exp	PBE	PW91	RPBE	BEEF-vdW	TPSS	SCAN	B3LYP	PBE0	r-MAE	r-MAX
$N_{2(g)} + CO_{(g)} + 4H^+ + 4e^- \rightarrow CO(NH_2)_{2(aq)}$	-0.69	-1.68	-1.71	-0.98	-0.80	-1.00	-1.79	-1.14	-1.92	0.69	1.23
$N_{2(g)} + CO_{2(g)} + 6H^+ + 6e^- \rightarrow CO(NH_2)_{2(aq)} + H_2O_{(l)}$	-0.48	-1.04	-1.10	-0.39	-0.20	-0.36	-1.25	-0.79	-1.48	0.47	1.00
$2NO_{(g)} + CO_{(g)} + 8H^+ + 8e^- \rightarrow CO(NH_2)_{2(aq)} + 2H_2O_{(l)}$	-7.42	-8.00	-8.22	-7.01	-6.76	-6.91	-8.15	-7.58	-8.47	0.61	1.04
$2NO_{(g)} + CO_{2(g)} + 10H^+ + 10e^- \rightarrow CO(NH_2)_{2(aq)} + 3H_2O_{(l)}$	-7.21	-7.36	-7.60	-6.42	-6.16	-6.28	-7.61	-7.23	-8.03	0.57	1.06
$2NO^{-}_{3(aq)} + CO_{(g)} + 16H^+ + 14e^- \rightarrow CO(NH_2)_{2(aq)} + 6H_2O_{(l)}$	-13.13	-11.88	-12.05	-11.23	-10.88	-11.06	-12.72	-12.92	-13.41	1.18	2.25
$2NO^{-}_{3(aq)} + CO_{2(g)} + 18H^+ + 16e^- \rightarrow CO(NH_2)_{2(aq)} + 7H_2O_{(l)}$	-12.92	-11.24	-11.44	-10.64	-10.28	-10.42	-12.18	-12.56	-12.97	1.47	2.64
xc-MAE	-	0.87	0.90	0.96	1.17	1.07	0.69	0.25	0.73		
xc-MAX	-	1.68	1.48	2.28	2.64	2.50	1.09	0.45	1.23		

Supplementary note 4: Thermal contributions

The DFT ground-state energy of a compound is given at 0 K, so in order to compute the respective energies at 298.15 K one may account for the thermal contribution from 0 to 298.15 K. This enthalpic contribution is shown in Equation 1 of the main text for a compound *i* as the difference between its formation enthalpy at 298.15 and 0 K, $\Delta_f \boldsymbol{H}_{\boldsymbol{i},@\mathbf{298.15}\,\boldsymbol{K}} - \Delta_f \boldsymbol{H}_{\boldsymbol{i},@\mathbf{0}\,\boldsymbol{K}}$. If the formation enthalpies are unknown, the enthalpic contributions in a chemical reaction can be estimated as the difference between the sum of the integral of the specific heats ($\boldsymbol{C_p}$) of reactants (R) and products (P) from 0 to 298.15 K multiplied by their stoichiometric coefficients, i.e., $\Delta_r \boldsymbol{H}_{\boldsymbol{i},@\mathbf{298.15}\,\boldsymbol{K}} - \Delta_r \boldsymbol{H}_{\boldsymbol{i},@\mathbf{0}\,\boldsymbol{K}} \approx \sum_{i=1}^{P} \int_0^{298.15K} \boldsymbol{C}_{\boldsymbol{p},\boldsymbol{i}} - \sum_{j=1}^{R} \int_0^{298.15K} \boldsymbol{C}_{\boldsymbol{p},\boldsymbol{j}}$. These quantities can be estimated from the vibrational frequencies and using the ideal gas limit approximation.[12] Supplementary Table 5 contains the formation enthalpies of all molecules in this study at 298.15 and 0 K, and their difference.

Supplementary Table 5. Thermal contributions of the gaseous species in this study. All values are in eV and taken from ref.[2]

molecule	$\Delta_f H_{@0\,K}$	$\Delta_f H_{@298.15\,K}$	$\Delta_f H_{@298.15\,K} - \Delta_f H_{@0\,K}$
C	-	-	0.01
O_2	0.000	0.000	0.000
H_2	0.000	0.000	0.000
N_2	0.000	0.000	0.000
CO_2	-4.075	-4.078	-0.004
Urea*	-2.287	-2.457	-0.170
HNO_3	-1.287	-1.388	-0.101
H_2O*	-2.476	-2.506	-0.030
NH_3	-0.404	-0.476	-0.072
NO	0.938	0.944	0.005
CO	-1.180	-1.146	0.034

*The values for aqueous and gaseous states are the same as the former is obtained from the latter.

S4.1 Effects on the gas-phase errors

When the aforementioned thermal corrections are low and/or their difference is small (e.g., lower than 0.10 eV), they are usually neglected when estimating the DFT free energy.[13,14] As the DFT-calculated formation energy is used in the estimation of the error of a gaseous compound, using Equation 12 in the main text (Equation S3 in this document), including or not the enthalpic contributions shifts the calculated DFT gas-phase error. In other words, if the enthalpic

contributions are not included in Equation 12, the gas-phase errors will implicitly account for that thermal correction. Anyway, gas-phase errors can be used to obtain DFT energies that match the experiments if consistency between Equations 1 and 12 is guaranteed.

If the enthalpic corrections are neglected in Equation 1, the resulting errors are shifted by the values in Supplementary Table 6. Notably, only the errors in N_2 and NO are shifted by more than 0.1 eV, while that of urea remains virtually unchanged.

Supplementary Table 6. Difference between the gas-phase errors calculated with and without enthalpic contributions from 0 to 298.15 K. Only the errors of N_2 and NO changed by more than 0.1 eV.

molecule	PBE	PW91	RPBE	BEEF-vdW	TPSS	SCAN	B3LYP	PBE0
N_2(g)	0.14	0.14	0.14	0.14	0.14	0.14	0.14	0.14
O_2(g)	0.06	0.06	0.06	0.06	0.06	0.06	0.06	0.06
urea (g)	-0.01	-0.01	-0.01	-0.01	-0.01	-0.01	-0.01	-0.01
urea (aq)	-0.01	-0.01	-0.01	-0.01	-0.01	-0.01	-0.01	-0.01
NO(g)	0.11	0.11	0.11	0.11	0.11	0.11	0.11	0.11
CO(g)	0.05	0.05	0.05	0.05	0.05	0.05	0.05	0.05
CO_2(g)	0.05	0.05	0.05	0.05	0.05	0.05	0.05	0.05
HNO_3(g)	0.06	0.06	0.06	0.06	0.06	0.06	0.06	0.06
NO_3^-(aq)	0.06	0.06	0.06	0.06	0.06	0.06	0.06	0.06

Supplementary note 5: Free-energy diagrams

The free energy diagrams of the co-electrolysis reactions (Equations 2 to 6 in the main text) on the thermodynamically ideal catalyst are shown in Supplementary Figures 3-7. The plot for the co-electroreduction of $\boldsymbol{NO_{3(aq)}^-}$and $CO_{2(g)}$ to urea is shown in Figure 4 of the main text.

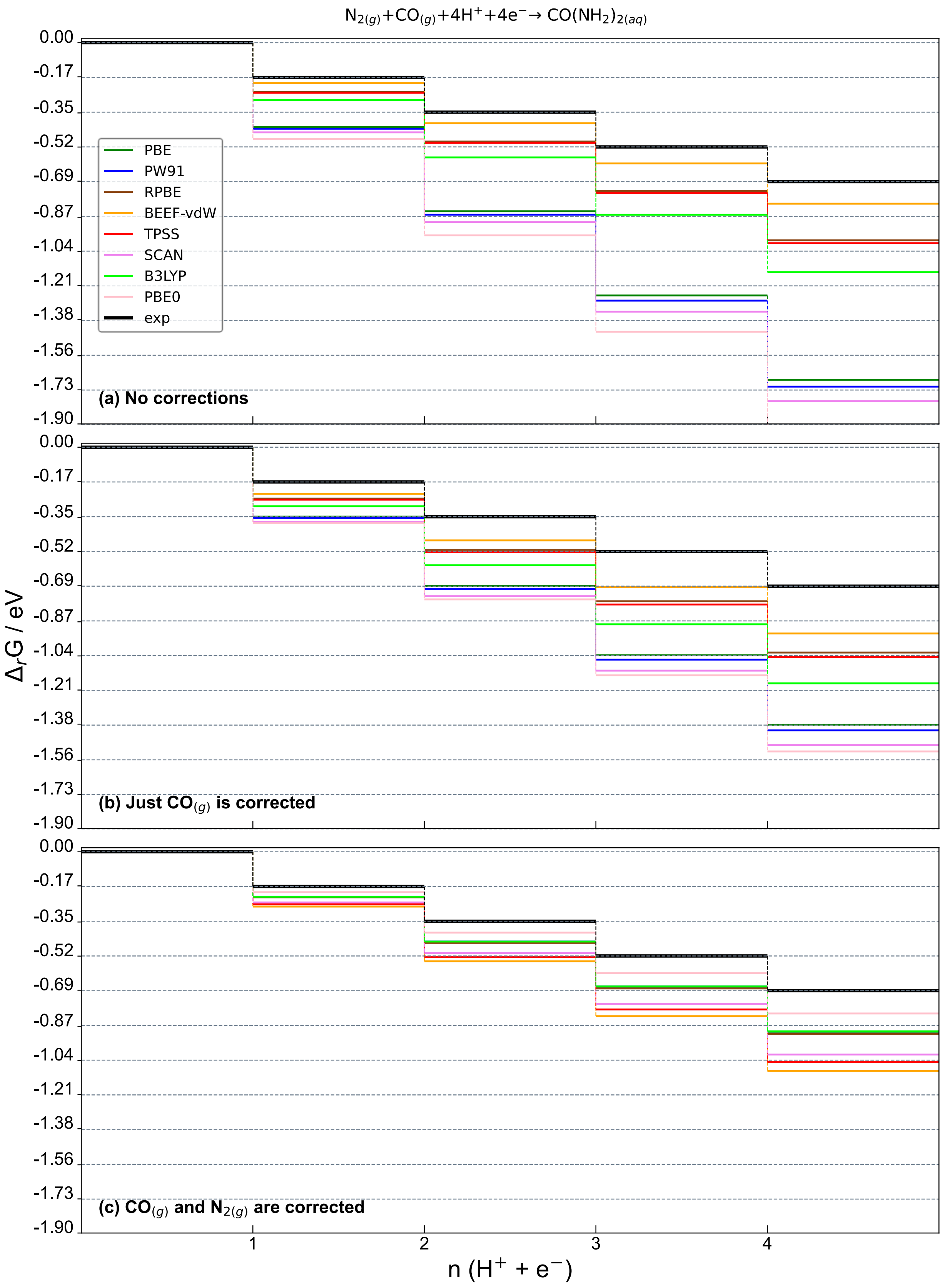

Supplementary Figure 3. Free-energy diagram of the co-electrolysis of $N_{2(g)}$ and $CO_{(g)}$ to urea on the thermodynamically ideal catalyst.

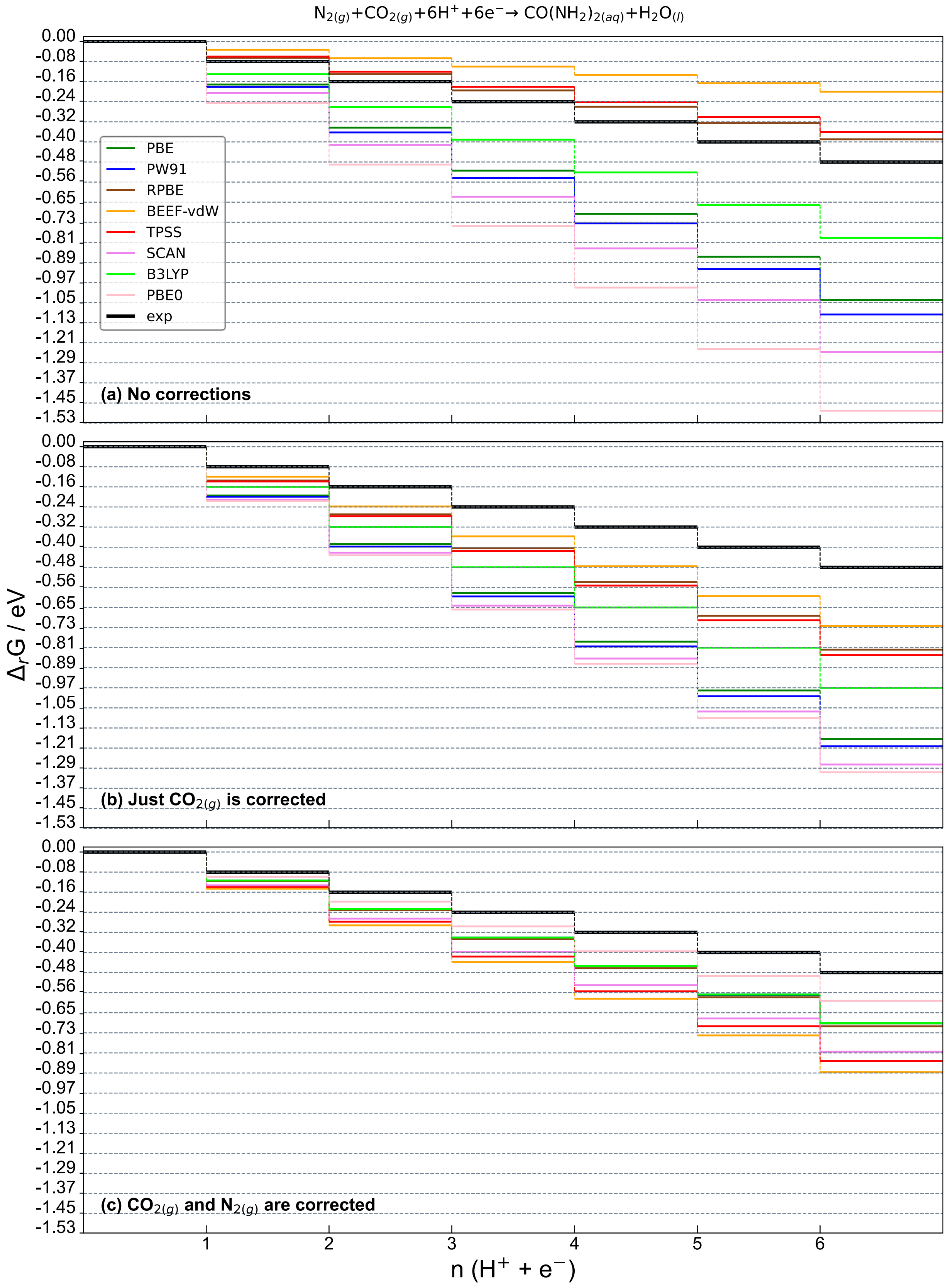

Supplementary Figure 4. Free-energy diagram of the co-electrolysis of $N_{2(g)}$ and $CO_{2(g)}$ to urea on the thermodynamically ideal catalyst.

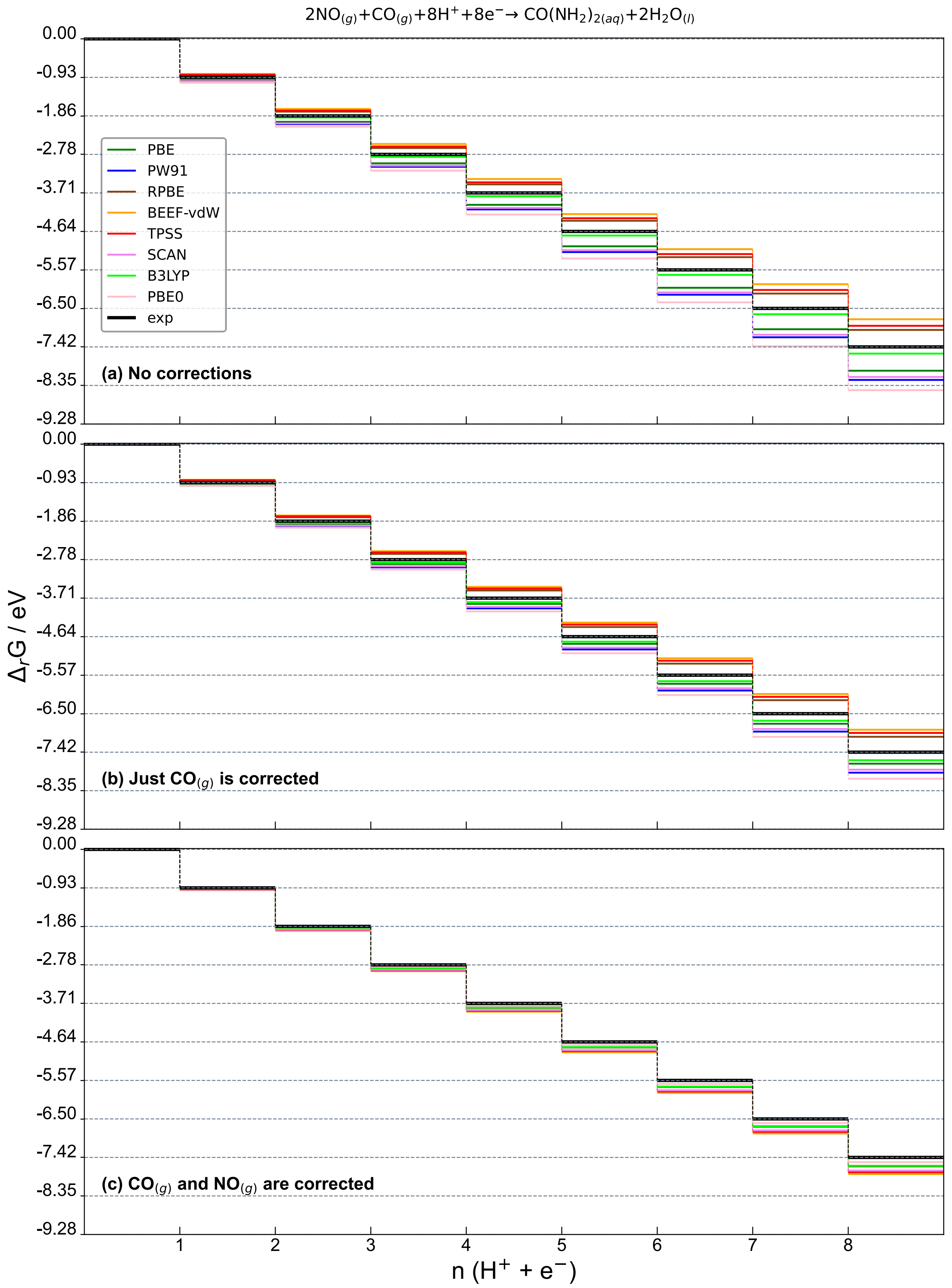

Supplementary Figure 5. Free-energy diagram of the co-electrolysis of $NO_{(g)}$ and $CO_{(g)}$ to urea on the thermodynamically ideal catalyst.

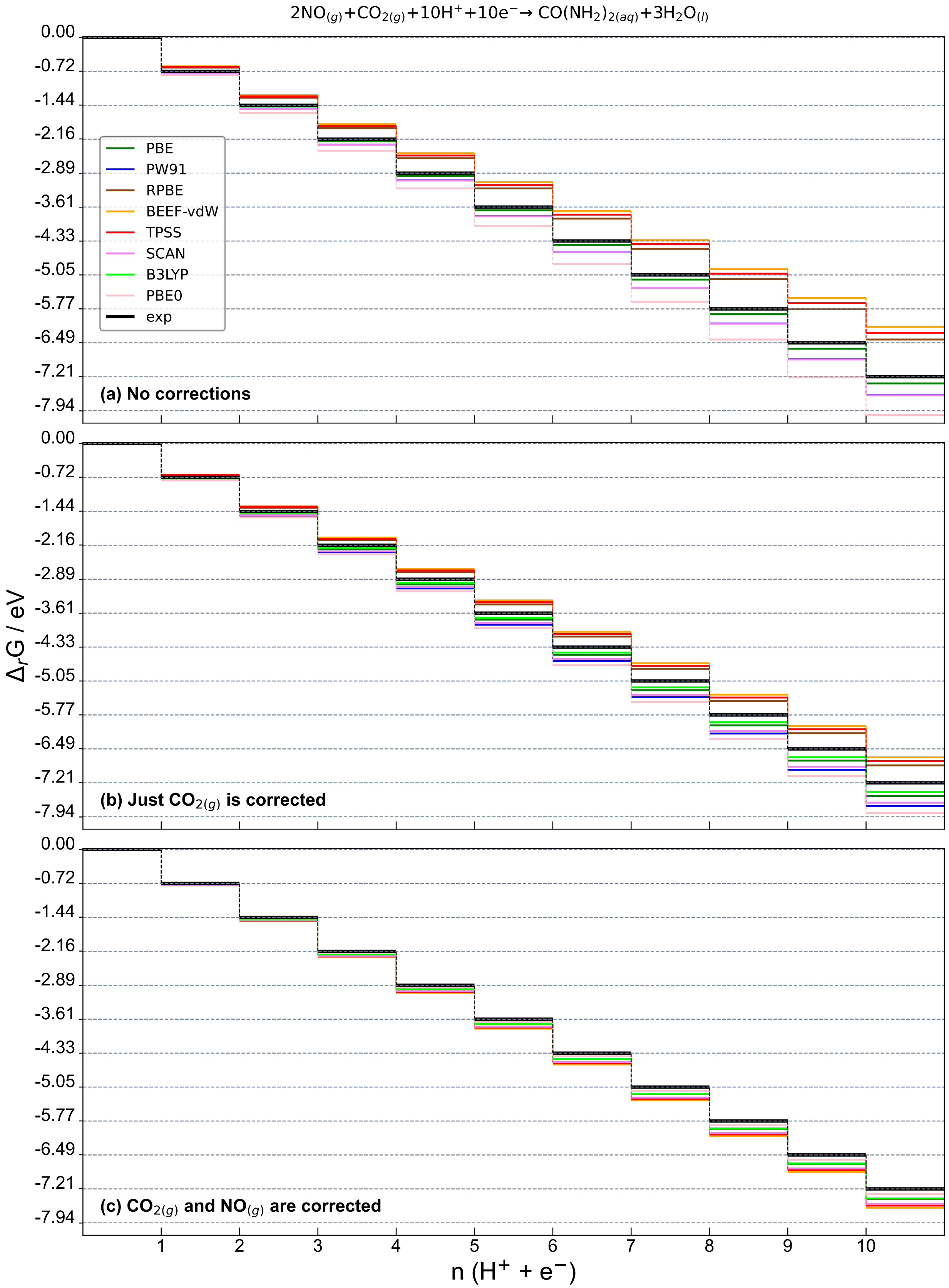

Supplementary Figure 6. Free-energy diagram of the co-electrolysis of $NO_{(g)}$ and $CO_{2(g)}$ to urea on the thermodynamically ideal catalyst.

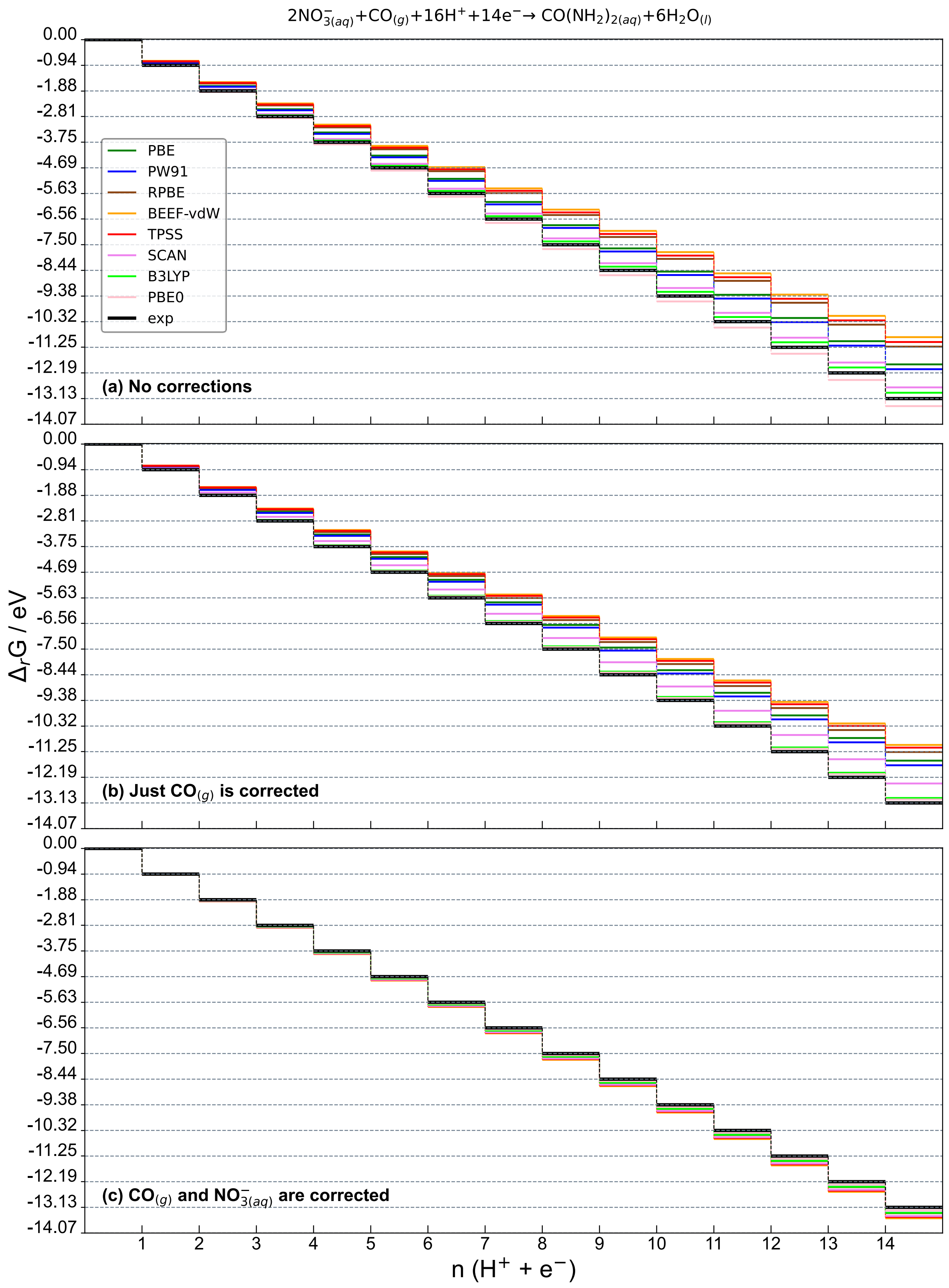

Supplementary Figure 7. Free-energy diagram of the co-electrolysis of $NO^{-}_{3(aq)}$ and $CO_{(g)}$ to urea on the thermodynamically ideal catalyst.

Supplementary note 6: Alternative thermodynamic cycles

Alternative routes to estimate the energies of ionic species in solution exist that make use of experimental equilibrium potentials. For instance, following a simple thermodynamic cycle in the light of previous works,[15,16] here we show how the energy of nitrate ($NO^-_{3(aq)}$) can be obtained from the energies of $N_{2(g)}$, $H_2O_{(l)}$, and $H_{2(g)}$. First, consider the reduction reaction of nitrate to $N_{2(g)}$:

$$2NO^-_{3(aq)} + 12H^+ + 10e^- \rightarrow N_{2(g)} + 6H_2O_{(l)} \quad \text{(S10)}$$

The equilibrium potential (U^0) of this reaction is 1.244 V. The free energy of this reaction ($\Delta_r G$) can be expressed as follows.

$$\Delta_r G = G_{N_{2(g)}} + 6 \cdot G_{H_2O_{(l)}} - 2 \cdot G_{NO^-_{3(aq)}} - 12 \cdot G_{H^+} + 10\, eU \quad \text{(S11)}$$

If $U = U^0$, the reaction is in equilibrium and Equation S11 yields:

$$G_{NO^-_{3(aq)}} = (G_{N_{2(g)}} + 6 \cdot G_{H_2O_{(l)}} - 12 \cdot G_{H^+} + 10\, eU^o_{SHE})/2 \quad \text{(S12)}$$

Recalling that $G_{H_2O_{(l)}} = G_{H_2O_{(g)}} - 0.09\, eV$ and $12 \cdot G_{H^+} = 6\, G_{H_{2(g)}}$ by virtue of the CHE model,[6] Equation S12 calculates the energy of $NO^-_{3(aq)}$ from energies of species straightforwardly calculated with DFT. However, we note that gas-phase errors still play a role in this route, as the error of dinitrogen must be assessed, analogous to the thermodynamic cycle presented in the main text where $HNO_{3(g)}$ is used as a reference. If the error in dinitrogen is corrected, the free energy of nitrate obtained with Equation S12 can be used in Equation S2 to exactly reproduce its experimental formation free energy, provided that O_2 is also corrected. Finally, the same analysis would have been performed for aqueous urea if experimental equilibrium potentials were known.

Supplementary note 7: Calculation settings

S7.1 INCAR

The basic INCAR file used to carry a spin-restricted calculation is shown below for PBE. For the other xc-functionals, the GGA flag value was replaced following the guidelines of the VASP manual.[17]

```
SYSTEM = spin-restricted calculation
PREC = Normal
ICHARG = 2
ISPIN  = 1
ENCUT  = 450
NELMIN = 6
ISMEAR =0
```

SIGMA = 0.001
IBRION = 2
NSW = 480
POTIM = 0.25
EDIFF = 0.00001
EDIFFG = -0.01
LWAVE = F
LCHARG = F
GGA = PE

S7.2 KPOINTS

The KPOINTS file contains only the gamma point in a Monkhorst-Pack mesh,[18] as described in the Methods section.

S7.3 POTCAR

The POTCARs used were PAW_PBE 08Apr2002 for C, PAW_PBE 15Jun2001 for H, PAW_PBE 08Apr2002 for N, and PAW_PBE 08Apr2002 for O.

Supplementary note 8: Direct coordinates

The coordinates of the relaxed geometries for all the compounds studied in this work obtained using the SCAN xc-functional are shown below.

CO2
```
  1.00000000000000
   15.0000000000000000    0.0000000000000000    0.0000000000000000
    0.0000000000000000   15.0000000000000000    0.0000000000000000
    0.0000000000000000    0.0000000000000000   15.0000000000000000
  C    O
    1     2
Selective dynamics
Direct
  0.3524151333333307  0.3526384484094862  0.5634795305031801   T   T   T
  0.3524151333333307  0.3526374117857145  0.6411230445798839   T   T   T
  0.3524151333333307  0.3526362064714519  0.4858362249169385   T   T   T
```

CO
```
1.00000000000000
15.0000000000000000    0.0000000000000000    0.0000000000000000
0.0000000000000000   15.0000000000000000    0.0000000000000000
0.0000000000000000    0.0000000000000000   15.0000000000000000
C    O
    1     1
Selective dynamics
Direct
  0.3524151333333307  0.3524151333333307  0.5657279182512692   T   T   T
  0.3524151333333307  0.3524151333333307  0.6412972150820625   T   T   T
```

H2
```
  1.00000000000000
   15.0000000000000000    0.0000000000000000    0.0000000000000000
    0.0000000000000000   15.0000000000000000    0.0000000000000000
    0.0000000000000000    0.0000000000000000   15.0000000000000000
  H
    2
Selective dynamics
Direct
  0.3524151333333307  0.3524151333333307  0.5637804204692849   T   T   T
  0.3524151333333307  0.3524151333333307  0.6132447128640457   T   T   T
```

H2O
```
  1.00000000000000
   15.0000000000000000    0.0000000000000000    0.0000000000000000
    0.0000000000000000   15.0000000000000000    0.0000000000000000
    0.0000000000000000    0.0000000000000000   15.0000000000000000
  H    O
    2     1
Selective dynamics
Direct
  0.0000000000000000  0.0509757900522118  0.9687447059740469   T   T   T
 -0.0000000000000000  0.9490242099477884  0.9687447059740469   T   T   T
  0.0000000000000000  0.0000000000000000  0.0077705880518965   T   T   T
```

HNO3
```
  1.00000000000000
   15.0000000000000000    0.0000000000000000    0.0000000000000000
    0.0000000000000000   15.0000000000000000    0.0000000000000000
    0.0000000000000000    0.0000000000000000   15.0000000000000000
  H    N    O
    1     1     3
Selective dynamics
Direct
  0.0413253640036283  0.8915938792721499  0.0000000000000000   T   T   T
  0.0000394898791012  0.0106301077598093  0.0000000000000000   T   T   T
  0.9818954283483934  0.9178194799643464  0.0000000000000000   T   T   T
  0.0785988827837178  0.0311278066409641  0.0000000000000000   T   T   T
  0.9342608349851638  0.0565687263627346  0.0000000000000000   T   T   T
```

N2

```
  1.00000000000000
   15.0000000000000000    0.0000000000000000    0.0000000000000000
    0.0000000000000000   15.0000000000000000    0.0000000000000000
    0.0000000000000000    0.0000000000000000   15.0000000000000000
  N
    2
Selective dynamics
Direct
  0.3524151333333307  0.3524151333333307  0.5666312487637978   T   T   T
  0.3524151333333307  0.3524151333333307  0.6403938845695339   T   T   T
```

NH3

```
  1.00000000000000
   15.0000000000000000    0.0000000000000000    0.0000000000000000
    0.0000000000000000   15.0000000000000000    0.0000000000000000
    0.0000000000000000    0.0000000000000000   15.0000000000000000
  N    H
    1     3
Selective dynamics
Direct
  0.0000000000000000  0.9999881238204535 -0.0001267836404722   T   T   T
  0.0000000000000000  0.9372521574444199  0.9746017969693390   T   T   T
  0.0543388334330723  0.0313831927009024  0.9746024933355654   T   T   T
  0.9456611665669277  0.0313831927009024  0.9746024933355654   T   T   T
```

NO

```
  1.00000000000000
   15.0000000000000000    0.0000000000000000    0.0000000000000000
    0.0000000000000000   15.0999999999999996    0.0000000000000000
    0.0000000000000000    0.0000000000000000   15.1999999999999993
  O    N
    1     1
Selective dynamics
Direct
  0.3524151333333307  0.3500812582781450  0.6337717917134477   T   T   T
  0.3524151333333307  0.3500812582781450  0.5573714319707598   T   T   T
```

O2

```
  1.00000000000000
   15.0000000000000000    0.0000000000000000    0.0000000000000000
    0.0000000000000000   15.0000000000000000    0.0000000000000000
    0.0000000000000000    0.0000000000000000   15.0000000000000000
  O
    2
Selective dynamics
Direct
 -0.0000000000000000  0.0000000000000000 -0.0001577914863325   T   T   T
  0.0000000000000000  0.0000000000000000  0.0814911248196657   T   T   T
```

CO(NH2)2

```
  1.00000000000000
   15.0000000000000000    0.0000000000000000    0.0000000000000000
    0.0000000000000000   15.0000000000000000    0.0000000000000000
    0.0000000000000000    0.0000000000000000   15.0000000000000000
  N    H    C    O
    2     4     1     1
Selective dynamics
Direct
  0.9205467048184276  0.0105808146715186  0.9637216851734893   T   T   T
  0.0725732966940445  0.9846383455164970  0.9588992411828231   T   T   T
  0.9245404400505401  0.0384010853741423  0.9026926332549585   T   T   T
  0.8689757710790331  0.0305913016794717  0.0018130174676722   T   T   T
  0.0658600985195516  0.9638657366590249  0.8953248899300779   T   T   T
  0.1252385244443219  0.9594823263158206  0.9921462492045298   T   T   T
  0.9975290521907229  0.9942881726751222  0.0107333932806072   T   T   T
  0.9992027788700435  0.9889522171084072  0.0919355571724964   T   T   T
```

Supplementary references

(1) Linstrom, P. J.; Mallard, W. G. NIST Chemistry WebBook, NIST Standard Reference Database 69. https://doi.org/10.18434/T4D303.

(2) *NIST Computational Chemistry Comparison and Benchmark Database*. NIST Standard Reference Database 101. https://cccbdb.nist.gov/introx.asp (accessed 2022-05-15).

(3) Linstrom, P. J. *NIST Chemistry WebBook - SRD 69, National Institute of Standards and Technology*. https://webbook.nist.gov/chemistry (accessed 2023-05-17).

(4) Kurth, S.; Perdew, J. P.; Blaha, P. Molecular and Solid-State Tests of Density Functional Approximations: LSD, GGAs, and Meta-GGAs. *Int. J. Quantum Chem.* **1999**, *75* (4-5), 889–909. https://doi.org/10.1002/(SICI)1097-461X(1999)75:4/5<889::AID-QUA54>3.0.CO;2-8.

(5) Urrego-Ortiz, R.; Builes, S.; Calle-Vallejo, F. Fast Correction of Errors in the DFT-Calculated Energies of Gaseous Nitrogen-Containing Species. *ChemCatChem* **2021**, *13* (10), 2508–2516. https://doi.org/10.1002/cctc.202100125.

(6) Nørskov, J. K.; Rossmeisl, J.; Logadottir, A.; Lindqvist, L.; Kitchin, J. R.; Bligaard, T.; Jónsson, H. Origin of the Overpotential for Oxygen Reduction at a Fuel-Cell Cathode. *J. Phys. Chem. B* **2004**, *108* (46), 17886–17892. https://doi.org/10.1021/jp047349j.

(7) Sargeant, E.; Illas, F.; Rodríguez, P.; Calle-Vallejo, F. Importance of the Gas-Phase Error Correction for O2 When Using DFT to Model the Oxygen Reduction and Evolution Reactions. *J. Electroanal. Chem.* **2021**, *896*, 115178. https://doi.org/10.1016/j.jelechem.2021.115178.

(8) Almeida, M. O.; Kolb, M. J.; Lanza, M. R. V.; Illas, F.; Calle-Vallejo, F. Gas-Phase Errors Affect DFT-Based Electrocatalysis Models of Oxygen Reduction to Hydrogen Peroxide. *ChemElectroChem* **2022**, *9* (12), e20220021 (1-7). https://doi.org/10.1002/celc.202200210.

(9) Haynes, W. M.; Lide, D. R.; Bruno, T. J. *CRC Handbook of Chemistry and Physics*, 97th ed.; CRC Press/Taylor And Francis: Boca Raton, FL, 2016. https://doi.org/10.1201/9781315380476.
(10) Granda-Marulanda, L. P.; Rendón-Calle, A.; Builes, S.; Illas, F.; Koper, M. T. M.; Calle-Vallejo, F. A Semiempirical Method to Detect and Correct DFT-Based Gas-Phase Errors and Its Application in Electrocatalysis. *ACS Catal.* **2020**, *10* (12), 6900–6907. https://doi.org/10.1021/acscatal.0c01075.
(11) Urrego-Ortiz, R.; Builes, S.; Calle-Vallejo, F. Impact of Intrinsic Density Functional Theory Errors on the Predictive Power of Nitrogen Cycle Electrocatalysis Models. *ACS Catal.* **2022**, *12* (8), 4784–4791. https://doi.org/10.1021/acscatal.1c05333.
(12) Cramer, C. J. *Essentials of Computational Chemistry: Theories and Models*, 2nd ed.; Wiley: Chichester, West Sussex, England ; Hoboken, NJ, 2004.
(13) Urrego-Ortiz, R.; Builes, S.; Calle-Vallejo, F. Automated versus Chemically Intuitive Deconvolution of Density Functional Theory (DFT)-Based Gas-Phase Errors in Nitrogen Compounds. *Ind. Eng. Chem. Res.* **2022**, *61* (36), 13375–13382. https://doi.org/10.1021/acs.iecr.2c02111.
(14) Bartel, C. J.; Weimer, A. W.; Lany, S.; Musgrave, C. B.; Holder, A. M. The Role of Decomposition Reactions in Assessing First-Principles Predictions of Solid Stability. *Npj Comput. Mater.* **2019**, *5* (1), 4. https://doi.org/10.1038/s41524-018-0143-2.
(15) Granda-Marulanda, L. P.; McCrum, I. T.; Koper, M. T. M. A Simple Method to Calculate Solution-Phase Free Energies of Charged Species in Computational Electrocatalysis. *J. Phys. Condens. Matter* **2021**, *33* (20), 204001. https://doi.org/10.1088/1361-648X/abf19d.
(16) Bondarenko, A. S.; Stephens, I. E. L.; Hansen, H. A.; Pérez-Alonso, F. J.; Tripkovic, V.; Johansson, T. P.; Rossmeisl, J.; Nørskov, J. K.; Chorkendorff, I. The Pt(111)/Electrolyte Interface under Oxygen Reduction Reaction Conditions: An Electrochemical Impedance Spectroscopy Study. *Langmuir* **2011**, *27* (5), 2058–2066. https://doi.org/10.1021/la1042475.
(17) *The VASP Manual*. https://www.vasp.at/wiki/index.php/The_VASP_Manual (accessed 2022-06-22).
(18) Monkhorst, H. J.; Pack, J. D. Special Points for Brillouin-Zone Integrations. *Phys. Rev. B* **1976**, *13* (12), 5188–5192. https://doi.org/10.1103/PhysRevB.13.5188.
